# Phage resistance at the cost of virulence: *Listeria monocytogenes serovar* 4b requires galactosylated teichoic acids for InlB-mediated invasion

**DOI:** 10.1371/journal.ppat.1008032

**Published:** 2019-10-07

**Authors:** Eric T. Sumrall, Yang Shen, Anja P. Keller, Jeanine Rismondo, Maria Pavlou, Marcel R. Eugster, Samy Boulos, Olivier Disson, Pierre Thouvenot, Samuel Kilcher, Bernd Wollscheid, Didier Cabanes, Marc Lecuit, Angelika Gründling, Martin J. Loessner

**Affiliations:** 1 Institute of Food, Nutrition and Health, ETH Zurich, Zurich, Switzerland; 2 Section of Microbiology and MRC Centre for Molecular Bacteriology and Infection, Imperial College London, London, United Kingdom; 3 Institute of Molecular Systems Biology, ETH Zurich, Zurich, Switzerland; 4 Biology of Infection Unit, Institut Pasteur, Paris, France; 5 Inserm U1117, Paris, France; 6 i3S - Instituto de Investigação e Inovação em Saúde; Institute for Molecular and Cell Biology, University of Porto, Porto, Portugal; 7 Paris Descartes University, Department of Infectious Diseases and Tropical Medicine, Necker-Enfants Malades University Hospital, APHP, Institut Imagine, Paris, France; Nanyang Technological University, SINGAPORE

## Abstract

The intracellular pathogen *Listeria monocytogenes* is distinguished by its ability to invade and replicate within mammalian cells. Remarkably, of the 15 serovars within the genus, strains belonging to serovar 4b cause the majority of listeriosis clinical cases and outbreaks. The *Listeria* O-antigens are defined by subtle structural differences amongst the peptidoglycan-associated wall-teichoic acids (WTAs), and their specific glycosylation patterns. Here, we outline the genetic determinants required for WTA decoration in serovar 4b *L*. *monocytogenes*, and demonstrate the exact nature of the 4b-specific antigen. We show that challenge by bacteriophages selects for surviving clones that feature mutations in genes involved in teichoic acid glycosylation, leading to a loss of galactose from both wall teichoic acid and lipoteichoic acid molecules, and a switch from serovar 4b to 4d. Surprisingly, loss of this galactose decoration not only prevents phage adsorption, but leads to a complete loss of surface-associated Internalin B (InlB),the inability to form actin tails, and a virulence attenuation *in vivo*. We show that InlB specifically recognizes and attaches to galactosylated teichoic acid polymers, and is secreted upon loss of this modification, leading to a drastically reduced cellular invasiveness. Consequently, these phage-insensitive bacteria are unable to interact with cMet and gC1q-R host cell receptors, which normally trigger cellular uptake upon interaction with InlB. Collectively, we provide detailed mechanistic insight into the dual role of a surface antigen crucial for both phage adsorption and cellular invasiveness, demonstrating a trade-off between phage resistance and virulence in this opportunistic pathogen.

## Author summary

*L*. *monocytogenes* is a Gram-positive, food-borne, intracellular pathogen that causes severe infection in susceptible individuals. Interestingly, almost all infections are caused by a subset of strains belonging to certain serovars featuring a complex glycosylation pattern on their cell surface. Using an engineered bacteriophage that specifically recognizes these modifications we selected for mutants that lost these sugars. We found that the resulting strains are severely deficient in invading host cells as we observed that a major virulence factor mediating host cell entry requires galactose decoration of the cell surface for its function. Without this galactose decoration, the strain represents a serovar not associated with disease. Altogether, we show a complex interplay between bacteriophages, bacteria, and the host, demonstrating that cellular invasiveness is dependent upon a serovar-defining structure, which also serves as a phage receptor.

## Introduction

*L*. *monocytogenes* is a Gram-positive, facultative intracellular pathogen capable of causing severe infection in susceptible individuals with a mortality rate of up to 30% [1,2]. For a long time, serotyping represented the primary diagnostic technique for *L*. *monocytogenes* to characterize strains beyond the species level, a practice based upon the serological reaction between specific antisera and surface antigen. This differentiation depends mainly upon the carbohydrate diversity within the cell wall that defines the O-antigens. Of the twelve identified serovars in the species, 1/2 and 4b are responsible for the vast majority of listeriosis cases [3], signifying that serovar may be clinically relevant.

Peptidoglycan-anchored cell wall teichoic acids (WTAs) can vary greatly in structure and composition and confer the structural basis of the O-antigens in many Gram-positive bacteria [4]. WTAs are known to be involved in regulation of cell morphology and division, autolytic activity, ion homeostasis, protection from host defenses and antibiotics, and may mediate host cell invasion and colonization [5]. In addition, the enzymes required for WTA biosynthesis have been proposed as novel antibiotic targets [6]. On that note, Tunicamycin-mediated inhibition of WTAs in both *S*. *aureus* and *L*. *monocytogenes* was recently shown to affect cell morphology, biofilm formation and virulence [7]. In *Listeria*, the peptidoglycan bound WTAs are comprised of 20–30 repeating units of ribitol phosphate, which can be subdivided into two classes, depending whether or not *N-*acetylglucosamine (GlcNAc) moieties are integrated within the backbone of the chain [8,9]. The WTA structure can be further modified with rhamnose, GlcNAc, glucose (Glc) or galactose (Gal) residues. We have recently described the diversity of *Listeria* WTAs in detail [8], relating a distinct structure to each serovar. However, little has been elucidated on the structure-function relationship of these complex glycans on the *Listeria* cell surface. This is in contrast to the membrane-anchored lipoteichoic acids (LTAs), which in *Listeria* consist of repeating units of glycerol-phosphate decorated with Gal and D-alanine, and demonstrate far less structural diversity than WTAs [10–12]. D-alanine esterification of LTA has been implicated in the regulation of autolysin function, the assimilation of metal cations and the overall maintenance of ion homeostasis [13]. In *L*. *monocytogenes*, the virulence factor InlB was reported to associate with LTA [14], but recent evidence suggests that LTA is not sufficient to localize InlB to the cell wall [15].

Correlating virulence and strain characteristics has long presented a significant, but difficult question to microbiologists. For *Listeria*, almost all listeriosis cases arise from serovar 1/2 and 4b strains, while the latter cause the majority of all cases and nearly all major outbreaks worldwide [16]. It is noteworthy that serovar 4b strains do not all belong to the same genetic lineage [3,17,18]. Yet, gene clusters responsible for teichoic acid biosynthesis in these strains have been strongly associated with infectious potential [19]. Together, the available evidence suggests that the WTA of serovar 4b may contribute directly to virulence. Previous studies have provided some evidence for this hypothesis. For instance, it was found that mutation of *gtcA* led to a loss of 4b antigenicity [20] through a decrease of Glc and Gal decoration on the GlcNAc component of the WTA backbone [21]. This structural modification led to moderately attenuated virulence and reduced cell-to-cell spread [22]. Loss of Gal from the WTA of a 4c strain led to attenuated plaque formation on cell monolayers, likely due to aberrant actin tail formation [23,24], while loss of rhamnose from a serovar 1/2 strain led to release of certain cell-wall associated proteins, including internalin B [25], and an increased susceptibility to antimicrobial peptides [26]. While WTA modifications have been previously implicated in virulence, it is not known whether this is the case for serovar 4b WTA.

Interestingly, much of our knowledge on WTA structure and its genetic basis has come from the analysis of phage-resistant mutants [5], as many *Listeria* phages adsorb to WTA of specific serovars [27,28]. Mutations in WTA biosynthesis genes can lead to small changes in the glycosylation pattern which can block phage binding. Identifying such mutations could promote an understanding of the nature of the specific phage ligand, the cell wall of the bacterium in general, and the biosynthetic process involved in production of that ligand. For example, phage resistance in serovar 1/2 strains caused by mutations in genes necessary for the addition of rhamnose and/or GlcNAc to the WTA backbone resulted in an altered serovar [29]. Overall, this indicates that phage predation contributes serovar diversity in *Listeria*, and that phages could be used as tools to target serovar-specific structures that mediate infection and dissemination in the host.

Using the highly invasive serovar 4b strain WSLC 1042 [30] and the broad host-range phage A511 [31], we isolated bacteriophage insensitive mutants (BIMs) lacking glycosyl substitutions on WTA, which were severely attenuated in cellular invasiveness. Based on this, we explored the possibility that the 4b-specific antigens contribute directly to cellular invasion. By using an engineered virulent mutant of the 4b-specific bacteriophage A500 [32–34], we isolated BIMs that had specifically lost the Gal decorations on WTA and LTA through mutation of a galactosyltransferase, converting the strain to serovar 4d. We attribute the invasion reduction to a loss of surface presentation of the virulence factor internalin B (InlB). We here show that it directly and specifically adheres to galactosylated teichoic acids presented by serovar 4b cells. As InlB facilitates entry into non-professional phagocytes through interaction with the ubiquitous cMet receptor [35,36], we explored the properties of this mutant in various cell types *in vitro* and *in vivo*. Altogether, our study demonstrates a direct relationship between phage predation, bacterial surface carbohydrate decoration and functionality of an essential *L*. *monocytogenes* virulence factor.

## Results

### Phage predation selects for teichoic acid glycosylation mutants with reduced invasion potential

**Fig 1:**
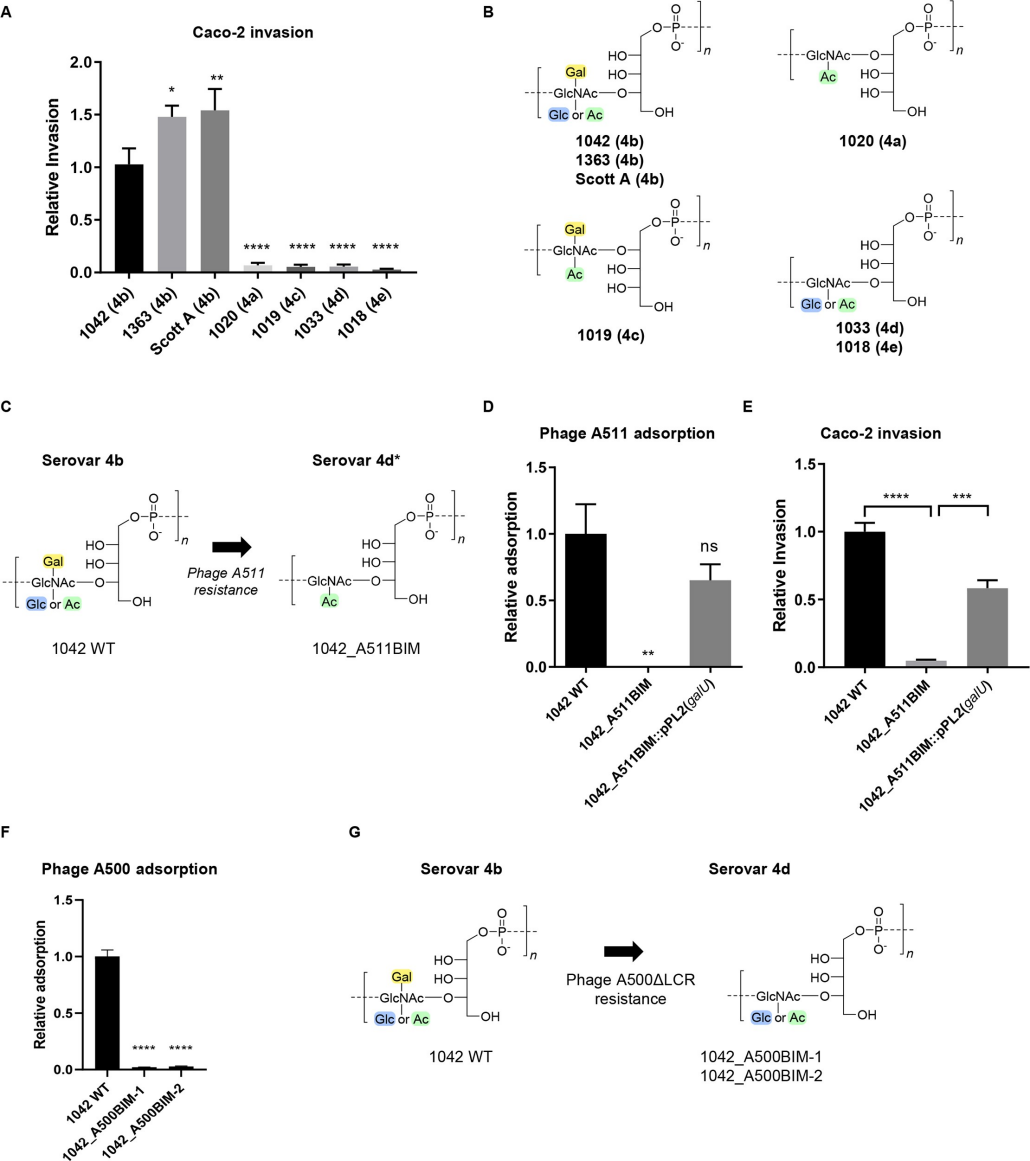
Epithelial cell invasiveness and phage resistance of *L*. *monocytogenes* strains with differing WTA structures. **(A)** Relative invasiveness of the indicated strains compared to 1042 WT determined by infecting Caco-2 cells for three hours (mean normalized to 1042 WT ± SEM; n = 4 for all strains except 1363 and ScottA, where n = 3) (all data is statistically significant from 1042 WT which serves as the serovar 4b control, as determined by a one-way ANOVA; ****P<0.0001; **P < 0.01; *P < 0.05; ns, not significant relative to 1042). **(B)** WTA monomer structures from strains of each serovar indicated in brackets, as determined by UPLC-MS/MS (representation of n = 3 experiments). **(C)** The change in WTA monomer structure of 1042_A511BIM selected for by challenge with phage A511, as determined by UPLC-MS/MS (data representative of two separate extractions) and the corresponding serovar designation. Color coding is provided for simplicity and clarification (yellow: galactose modification; blue: glucose modification; green: O-acetylaton). **(D)** Relative adsorption of phage A511 to 1042_A511BIM and 1042_A511BIM::pPL2(*galU*), compared to 1042 WT, as determined by phage pulldown assay (representation is mean of n = 3 experiments normalized to WT ± SEM; **P<0.01; ns, not significant relative to WT control). **(E)** Relative invasiveness of 1042_A511BIM and 1042_A511BIM::pPL2(*galU*) compared to 1042 WT of Caco-2 cells infected for three hours (error bars, SEM; n = 4; ****P<0.0001; ***P<0.001). **(F)** Relative adsorption of phage A500 WT to 1042 WT, 1042_A500BIM-1 and 1042_A500BIM-2, as determined by phage pulldown assay and CFU count (representation is mean adsorption of n = 3 experiments normalized as a fraction of WT ± SEM; ****P<0.0001; ns, not significant relative to WT control). Abbreviations: Gal, galactose; Glc, glucose; GlcNAc, *N*-acetylglucosamine; Ac, O-acetyl group. **(G)** The WTA monomer structures of BIMs selected for by phage A500ΔLCR challenge, as determined by UPLC-MS/MS (data representative of two separate extractions) and the corresponding serovar designation.

Within serogroup 4, strains belonging to serovar 4b have a clear advantage in their ability to invade cells of epithelial origin (Caco-2) over several other representative strains belonging to different serovars (**Fig 1A**), each possessing a different WTA structure (**Fig 1B**). This led to the hypothesis that the specific WTA structure found in 4b strains (**S1A Fig**) contributes to invasiveness. Given that glycosyl modifications on WTA were previously implicated as 4b-specific antigens [21], we hypothesized that these modifications may confer invasiveness.

WTA also functions as a phage receptor in Gram-positive bacteria. *Listeria* phage A511 is well-established as having a broad host range and can infect serovar 1/2 strains as well as 4b strains. After challenging strain 1042 in culture with phage A511, we isolated one single colony that survived attack by A511 and termed it 1042_A511BIM. WTA extraction and subsequent structural determination by UPLC-mass spectrometry (**Figs 1C and S1B**) revealed that the Gal and Glc decorations were absent from the GlcNAc residues incorporated into the WTA backbone, conferring a 4d-like serovar (4d*) (**see suppl. Table**) to this phage resistant strain (**Fig 1C**). By sequencing strain 1042_A511BIM, we discovered both a single-nucleotide polymorphism and a frame-shift in the gene *galU*, encoding a UDP-glucose pyrophosphorylase. Complementation of *galU* largely restored Gal and Glc decoration and phage A511 adsorption (**Figs 1D and S1B**). The phage-resistant strain 1042_A511BIM also demonstrated a significant decrease in invasion of Caco-2 cells, which was also restored upon *galU* complementation (**Fig 1E**).

To determine the specific roles of the Gal and Glc modifications, we investigated the genetic basis of WTA structure. The temperate phage A500 specifically infects 4b strains, but cannot infect 4d isolates, which differ merely by the absence of Gal on the WTA. Thus, we hypothesized that A500 requires Gal for adsorption, and thus loss of Gal results in phage resistance. Phage A500 initially presented a technical barrier due to its temperate lifestyle, meaning that it cannot be used to select for strains with cell wall modifications, as it would not exert enough selective pressure on the cell wall due to genomic integration and resulting homo-immunity.

With the aid of a recently established phage genome rebooting platform [37], the A500 genome was reassembled to exclude the entire lysogeny control region (LCR) (**S2A Fig**). The resulting phage demonstrated infection kinetics of a virulent (i.e. strictly lytic) virus (**S2C Fig**) and was unable to produce lysogens (**S2B Fig)**. This A500ΔLCR phage was then used to challenge the parental 1042 strain, from which we isolated two clones resistant to A500 adsorption (**Fig 1F**). Illumina re-sequencing revealed that one mutant harbored a point mutation in *galE*, encoding a UDP glucose-4-epimerase, and the other a single base substitution in a putative glycosyltransferase (**S2D Fig**). The putative glycosyltransferase, which we refer to as *gttA*, has 77% identity to *glcV*, reported to be involved in WTA galactosylation of a 4c strain [24], and also does not exist in SV 1/2 strains. The two BIMs harbored no other mutations in any ORFs. Mass spectrometric analysis of the WTA structure (**S2E Fig**) revealed that only the Gal but not Glc decoration was absent from the WTA, making the WTA produced by these two BIM strains identical to that of 4d (**Fig 1G**), a serovar not associated with high invasiveness.

### Teichoic acid galactose decoration is required for Caco-2 and HepG2 cell invasion

**Fig 2:**
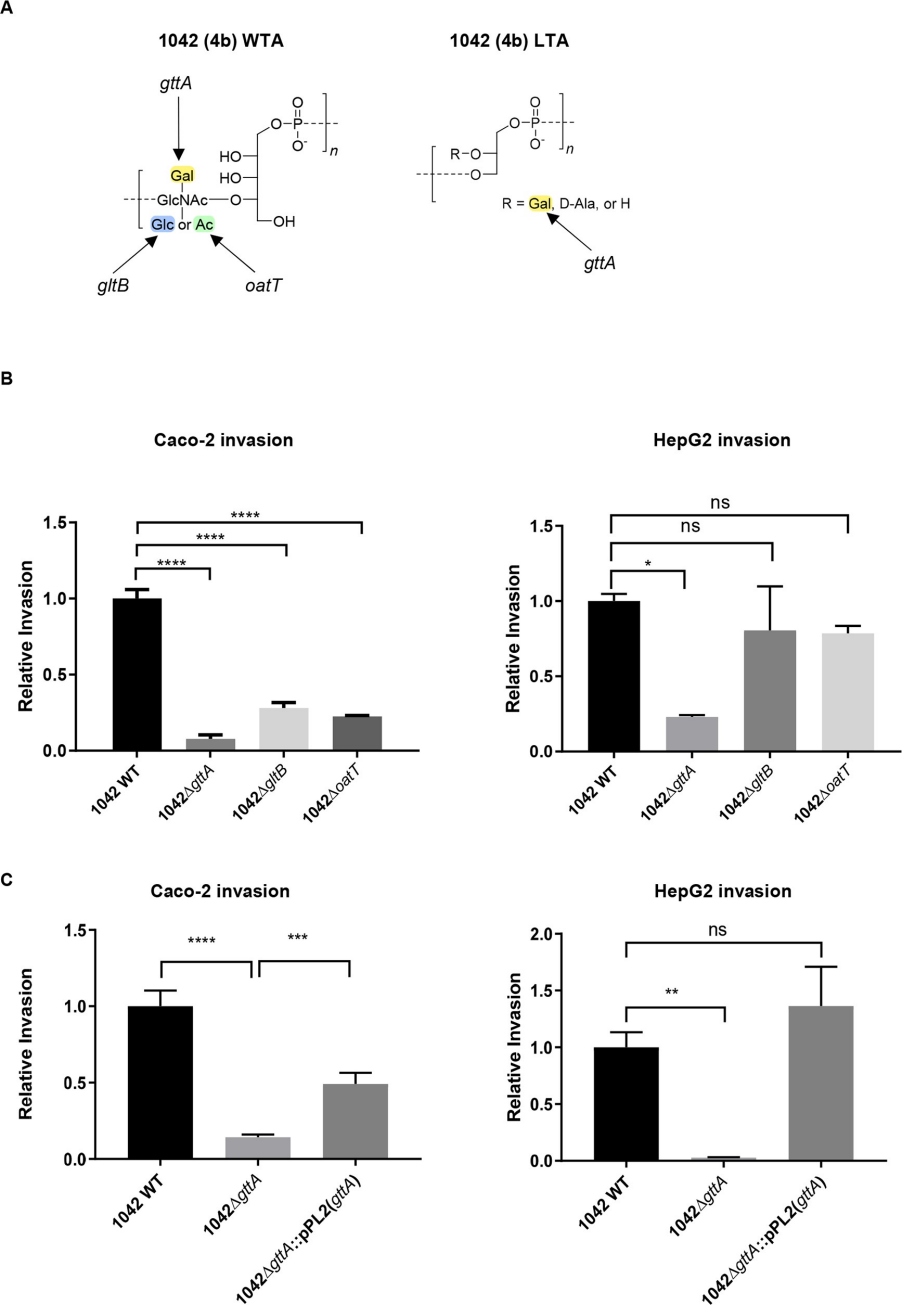
TA decoration and its effect on cellular invasiveness. **(A)** (left) A schematic representation of the WTA monomer structure of 1042 WT. Arrows indicate the individual structural decoration lost upon deletion of the indicated genes, as determined by MS analysis. (right) The LTA monomer structure and the resulting decoration loss following deletion of *gttA*, as determined by NMR analysis. **(B)** Relative invasiveness of the three mutants as in (A) to 1042 WT of Caco-2 cells infected for three hours (left: mean normalized to 1042 WT ± SEM; n = 4) and HepG2 cells (right: mean normalized to 1042 WT ± SEM; n = 3; ****P<0.0001; *P<0.05; ns = not significant relative to 1042 WT as determined by a one-way ANOVA). **(C)** Relative invasiveness of 1042Δ*gttA* and 1042Δ*gttA*::pPL2(*gttA*) complemented strain performed as in (B) (mean normalized to 1042 WT ± SEM; upper: n = 6; lower: n = 3; ****P<0.0001; ***P<0.001; **P<0.01). Abbreviations: Gal, galactose; Glc, glucose; GlcNAc, *N*-acetylglucosamine; Ac, O-acetyl group.

We wanted to further investigate whether the Gal decoration alone contributes to cellular invasiveness, given its exclusivity to serovar 4b. To test this hypothesis, we produced an in-frame knock-out of *gttA*, which again resulted in loss of WTA galactosylation (**Figs 2A and S3A**), and phage A500 adsorption (**S2F Fig**), and led to strain 1042Δ*gttA* being typed as 4d (**see suppl. Table**). Additionally, we deleted *gltB* [38], which expectedly led to a loss of Glc (**Figs 2A and S3A**), but did not significantly affect A500 adsorption (**S2F Fig**) and did not change serovar (**see suppl. Table**). Finally, following *in silico* examination of the WTA biosynthesis region in the 1042 genome (which harbors *gttA* and *galU*), we identified *oatT*, whose deletion led to the loss of WTA *O*-acetylation (**Figs 2A and S3A**), which did not affect A500 adsorption (**S2F Fig**). Overall, this set of three knockouts allowed us to further examine the effect of each respective modification of the serovar 4b WTA. We found that loss of glycosylation and *O*-acetylation did not change overall growth fitness (**S3B Fig**), but did result in a significantly higher sensitivity to the cephalosporin antibiotic cefotaxime (**S3C Fig**).

Since the LTA of *L*. *monocytogenes* is known to also feature a Gal decoration, we checked whether 1042Δ*gttA* had lost this modification as well. Previous studies have shown that the absence of sugar substitution on LTA leads to an increased western blot signal using a glycerol-phosphate specific primary antibody. Indeed, deletion of *gttA* resulted in a stronger signal (**S4A Fig**), and we confirmed the absence of Gal modification by NMR analysis (**Figs 2A and S4B)**, indicating that *gttA* is necessary for Gal decoration of both types of teichoic acid. We subsequently evaluated each deletion mutant for its ability to invade Caco-2 and HepG2 cells. Curiously, we observed that in Caco-2 cells, each modification seems to play a role in invasion, whereas in HepG2 cells, Gal appears to be the primary invasion mediator (**Fig 2B**), an effect restored upon *gttA* complementation (**Fig 2C)**. Further *in vitro* evaluation of the effect of teichoic acid galactosylation on cellular invasion by fluorescence microscopy showed a decrease in the percentage of Caco-2 cells infected within a monolayer, and a significantly decreased number of intracellular bacteria after three hours of infection (**S5A–S5C Fig**). We also observed that intracellular bacteria formed stunted actin tails (**S5D Fig**), as previously described following loss of Gal-WTA in a serovar 4c strain [24]. Altogether, these findings provide convincing evidence that loss of teichoic acid galactosylation, which bestows phage resistance, leads to an attenuated phenotype.

### Complementation with *gttA* transforms serovar 4d into 4b and restores invasiveness

**Fig 3:**
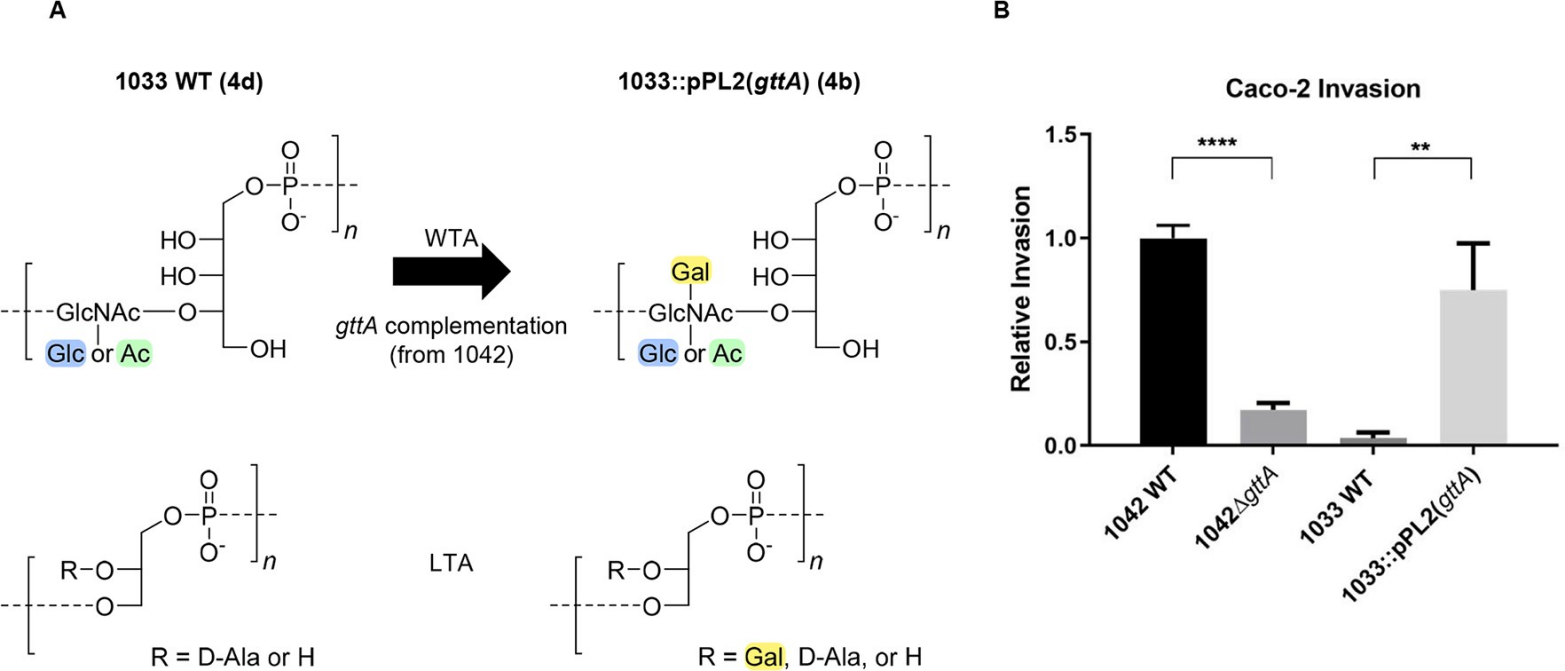
Heterologous *gttA* expression in the 4d strain 1033. **(A)** MS-based identification of WTA (upper) and NMR-based identification of LTA (lower) monomer residues from 1033 WT and the functional complement 1033::pPL2(*gttA*) along with the corresponding serovar designation. **(B)** Relative invasiveness of 1033 WT and 1033::pPL2(*gttA*) of Caco-2 cells infected for three hours of the same strains represented in (A) as compared to 1042 WT and 1042Δ*gttA* (mean ± SEM; n = 6; ****P<0.0001; **P<0.01).

Given that phage predation selected for a 4d mutant phenotype from a 4b background under laboratory conditions, we hypothesized that naturally occurring serovar 4d isolates, which are close genomic relatives of 4b strains, might have evolved in response to predation by a Gal-WTA-specific phage. WSLC 1033 is a 4d strain naturally resistant to phage A500 that shares 99.68% sequence homology to strain 1042 (**S2F Fig**). A sequence comparison of all known WTA biosynthesis genes between strains 1033 and 1042, revealed the only sequence difference to be a mutation in the *gttA* of strain 1033 leading to a glutamic acid deletion close to the *N*-terminus (**see suppl. Table**). Upon complementation of 1033 with functional *gttA* from strain 1042, the Gal decoration was reconstituted in both WTA and LTA (**Figs 3A, S4 and S6**). This changed the serovar to 4b, and significantly increased the strain’s invasiveness in cultured Caco-2 cells to levels similar to strain 1042 (**Fig 3B**). This rather interesting finding led us to theorize that 1033 may have derived from a previously invasive 4b strain that evolved in response to phage predation.

### Teichoic acid galactosylation is necessary for InlB cell wall association and cMet recognition

We wanted to investigate how galactosylated teichoic acids contribute to host cell invasion. In *S*. *aureus*, it was shown that WTA directly mediates adherence to endothelial cells [39]. In the case of *Listeria monocytogenes*, gene *gttA* is indeed required for adherence to Caco-2 and HepG2 cells (**S7A Fig**). However, competitive inhibition using purified WTA did not decrease adherence of *Listeria* to mammalian cells (**S7B Fig**), and WTA-coated latex beads did not exhibit any enhanced adherence relative to background (**S7C Fig**). This suggested that galactosylated WTAs by themselves do not directly mediate adherence to mammalian cells, but instead may regulate the function of a surface protein that functions as an adhesin. LTA was not investigated in this context due to the fact that it is buried in the peptidoglycan layer [12] and is therefore not presented for extracellular interaction.

**Fig 4:**
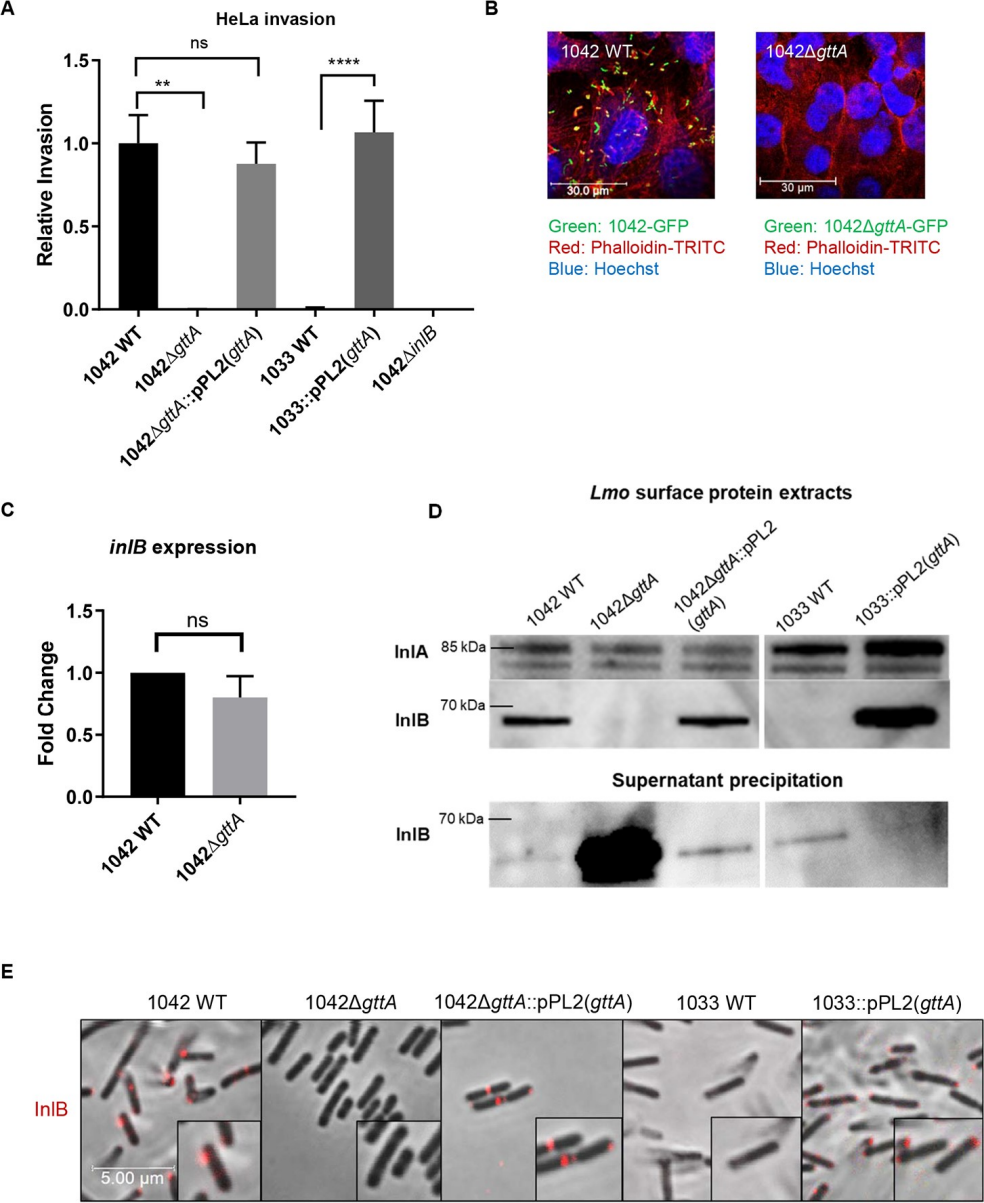
Galactosylated teichoic acids mediate InlB function and surface retention. **(A)** Relative invasiveness of the indicated strains relative to 1042 WT in HeLa cells, as determined by a three-hour infection assay (mean normalized to 1042 WT ± SEM; n = 3; ****P<0.0001; **P<0.01; ns = not significant relative to 1042 WT. Error bars on 1042Δ*gttA*, 1042Δ*inlB* and 1033 WT are too small to be properly depicted). **(B)** Fluorescence confocal microscopy of HeLa cells infected as in (B) with 1042 WT and 1042Δ*gttA* expressing GFP. Phalloidin-TRITC (red) stains actin and Hoechst (blue) stains DNA (images are representative from three individual experiments, contrast adjusted for clarity). **(C)** Quantitative real-time PCR analysis of *inlB* gene expression in the indicated strains (mean fold-change; error bars, SD; n = 3, ns = not significant). **(D)** Western blot of *Lmo* surface protein (upper) and precipitated supernatant (lower) of the indicated strains, detected using an anti-InlB antibody with anti-InlA as a loading control. Samples were normalized for OD and protein concentrations were further normalized before being loaded onto an SDS-PAGE gel (blot representative of three individual experiments). **(E)** Immunofluorescence of the indicated strains stained with an anti-InlB polyclonal antibody (Red) (scale bar applies to all images, images are representative of three experiments; contrast adjusted for clarity).

In HepG2 cells, it has been shown that invasion by *L*. *monocytogenes* is heavily dependent upon InlB, which possesses a membrane-spanning domain and extends through the bacterial cell wall. InlB’s primary function is to induce endocytosis by activating its receptor cMet, a mammalian receptor tyrosine kinase [36,40]. In HeLa cells, invasion is entirely InlB-dependent, because these cells do not express the Internalin A receptor, E-cadherin [41]. Strikingly, we observed a near-zero invasion rate of HeLa cells by strains lacking Gal on their TAs (**Fig 4A and 4B**), despite that *inlB* transcript levels did not change (**Fig 4C**). This suggests that InlB dysfunction may be responsible for the invasion defect observed in 1042Δ*gttA*.

**Fig 5:**
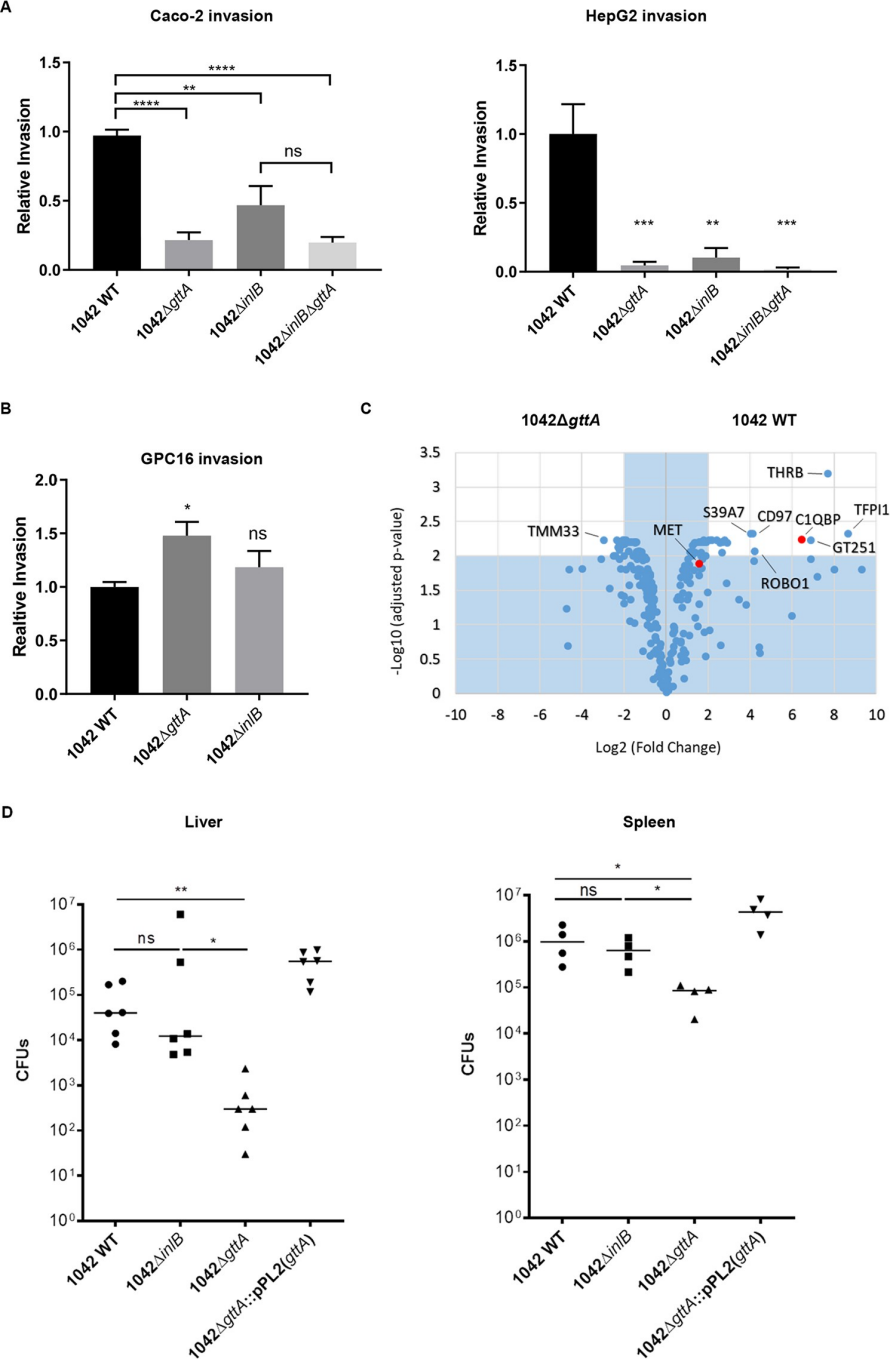
Galactosylated teichoic acids are required for cMet/gC1qR-mediated invasion and virulence. **(A)** Relative invasion of the indicated strains to 1042 WT following a three-hour infection of Caco-2 and HepG2 cells (mean ± SEM; for HepG2, n = 3; for Caco-2, n = 5; ****P<0.0001; ***P<0.001; **P<0.01, as determined by a one-way ANOVA; ns, not significant, as determined by a student’s t-test). **(B)** Relative invasion of the indicated strains to 1042 WT following infection of GPC16 cells (mean ± SEM; n = 6 from two individual experiments; *P<0.05; ns, not significant relative to WT). **(C)** HATRIC-LRC identification of target receptors of 1042 WT and 1042Δ*gttA* on the surface of HeLa cells. Relative fold changes of proteins (Log_2_ scale) are plotted against their respective log-transformed, false-discovery rate (FDR)-adjusted values. Target receptors are defined as proteins with a fold change greater than 4 and p-value less than or equal to 0.01, corresponding to the white space of the plot. Known receptors recognized by InlB are shown in red. **(D)** Bacterial burden in spleen and liver upon intravenous injection of *L*. *monocytogenes* and isogenic mutants. C57BL/6J mice were intravenously injected with 5x10^3^ CFUs/animal and dissected 3 days post infection. Values represent the number of CFUs per organ. One dot represents one animal; horizontal bar the median. Mann-Whitney tests were performed to determine statistical significance. **P<0.01, *P<0.05.

InlB possesses a C-terminal Csa domain containing several GW repeats, which has been shown to mediate association with cell-wall components such as LTA [14]. Differential teichoic acid display therefore may regulate InlB localization to the cell surface, especially given that it extends through, but is not covalently bound to the cell wall. Western blot analysis of *L*. *monocytogenes* surface protein extracts lacking Gal on their TAs showed a drastically decreased amount of InlB in the cell wall, and a significantly increased amount in the supernatant (although 1033 shows overall less, likely due to a lower level of InlB expression) (**Fig 4D and 4E**), suggesting that InlB is released from the cell surface. Complementation of 1042Δ*gttA* and 1033 with a functional copy of *gttA* restored the cell wall attachment of InlB (**Fig 4D and 4E**), indicating that the Gal modifications on the TAs are necessary for correct cell wall localization. The invasion rate of strain 1042Δ*inlB* in HepG2 and Caco-2 cells was strikingly similar to that of 1042Δ*gttA*; and a 1042Δ*gttA*Δ*inlB* double mutant did not demonstrate a synergistic effect, underlining that InlB function is abolished when Gal is missing from the TAs (**Fig 5A**). Surprisingly, invasion of GPC16 cells, which is InlB independent [42], actually increased slightly in 1042Δ*gttA* (**Fig 5B**). This indicates that the invasion attenuation in 1042Δ*gttA* is primarily mediated by InlB, and that InlA function is not attenuated. Next, a chemoproteomics approach [43] that allows for unbiased identification of target receptors on the surface of living cells (HATRIC-LRC) was performed to identify HeLa surface receptors involved in the binding of 1042 and 1042Δ*gttA*. Our findings revealed that only 1042 and not 1042Δ*gttA* cells associate with the known receptors cMet and gC1qR (C1QBP) (**Fig 5C**), supporting the notion that 1042Δ*gttA* cannot perform InlB-mediated invasion. HATRIC-LRC analysis using purified InlB protein taken from the 1042 genome sequence as a ligand revealed cMet as the major interacting partner, further supporting the validity of the approach, and that other HeLa receptors (THRB and CD97) enriched in the 1042 WT sample are not recognized by InlB, and therefore do not participate in invasion in this cell line (**S8A Fig**). Molecular inhibition of CD97 and THR revealed no effect on the invasion of 1042 in HeLa cells, in contrast to inhibition of Gc1qR, which as expected, did reveal an inhibitory effect (**S8B Fig**). Taken together, these results demonstrate that the loss of cell wall associated InlB due to Gal-deficient teichoic acid polymers is responsible for the decrease in cellular invasion.

Finally, we evaluated whether 1042Δ*gttA* demonstrated a virulence attenuation *in vivo*, which indeed showed a significantly lower CFU count relative to 1042 WT in the liver and spleen of intravenously-infected C57BL/6J mice (**Fig 5D**). However, consistent with what has been demonstrated in the literature (54), 1042Δ*inlB* showed only a very mild attenuation, demonstrating that loss of InlB function alone is clearly not the sole contributor to the 1042Δ*gttA* virulence attenuation, and likely also involves ActA dysfunction (**S5D Fig**).

**Fig 6:**
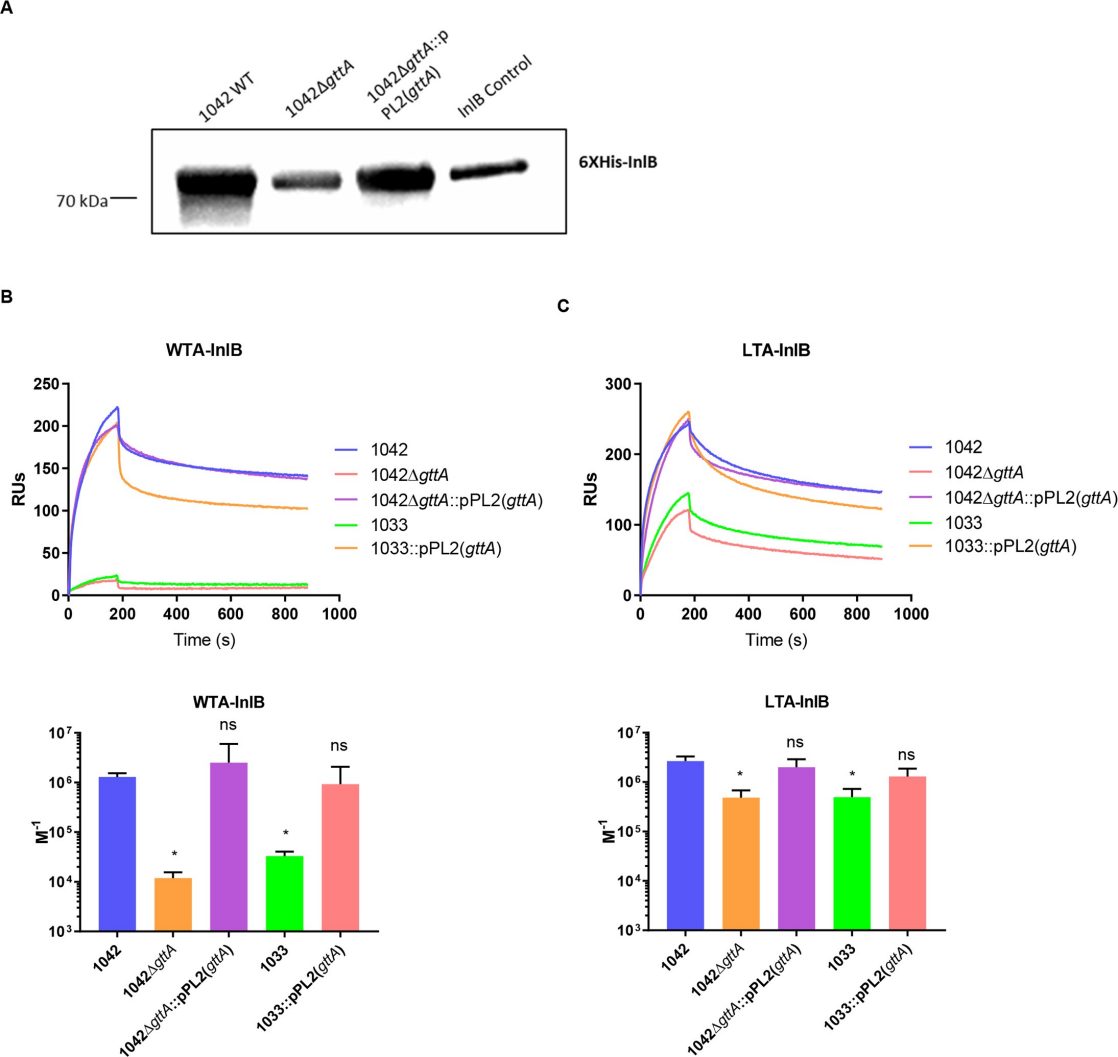
Interaction of purified InlB with the native cell wall, WTA and LTA. **(A)** Pulldown binding assay of His-tagged InlB to the indicated strains. WB of the entire *Listeria* pellet was performed, followed by detection using a His-tag antibody (control is 5 μg of purified His-tagged InlB only; blot is representative of three separate experiments, except for the complemented strain where n = 2). **(B)** Binding affinity response of immobilized InlB with WTA (upper) and the estimated K_A_ (lower) for each interaction, as determined via BIAcore analysis. (**C**) Binding affinity response of immobilized InlB with LTA (upper) and the estimated K_A_ (lower) for each interaction, as determined via BIAcore analysis. (RU: relative units; data for affinity responses of LTA/WTA is representative of three individual experiments, each time using newly purified InlB and WTA/LTA extracts). Estimated K_A_ was determined by averaging four replicate titrations (see figure S9B for example) using two different chips with immobilized InlB; error bars represent SD for the four replicates; significance was determined via a paired one-way ANOVA; *P<0.05, ns = not significant).

**Fig 7:**
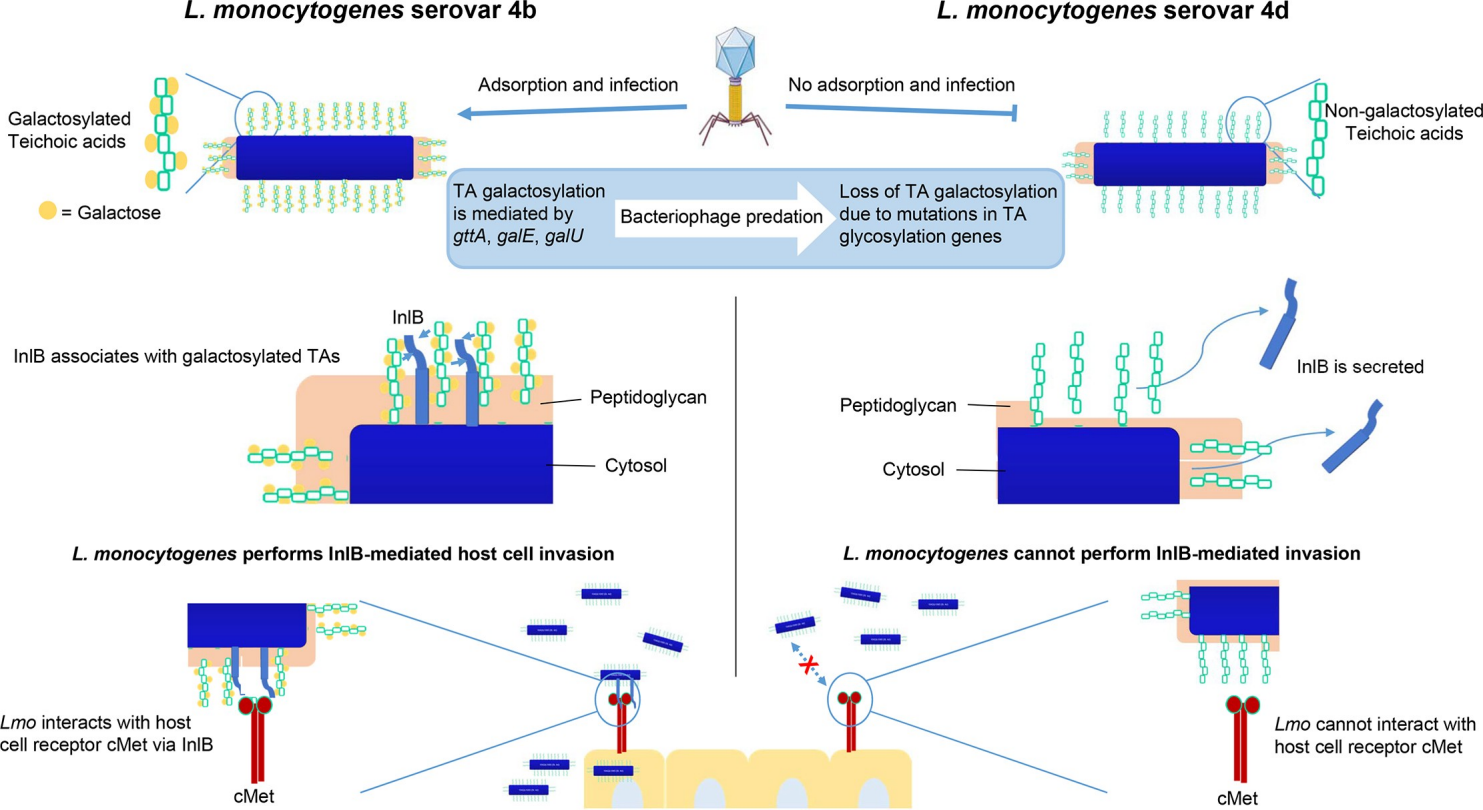
Schematic representation of the TA/InlB-mediated invasion mechanism. In *L*. *monocytogenes* serovar 4b, teichoic acids are galactosylated, and it is the galactosylated WTAs which confer the 4b serovar antigenicity. 4b strains are susceptible to bacteriophage predation due to the fact that broad host range bacteriophage (such as A511) and narrow host-range bacteriophage (such as 4b-specific A500) require the WTA galactose decoration for adsorption. Predation by these bacteriophages can lead to the selection of BIMs that have lost the galactose decoration via mutations in genes required for the biosynthesis of TA glycosylation *gttA*, *galE* or *galU*). These mutants are designated serovar 4d. The strain WSLC1033 is a WT 4d strain that harbors a mutation in *gttA*, and is therefore resistant to the 4b-specific phage A500. The serovar 4d knockout 1042Δ*gttA* lacks the TA galactose decoration which leads to a loss of InlB surface expression, due to the fact that InlB associates with TAs in a Gal-dependent manner. As a result, 1042Δ*gttA* cannot associate with cMet to activate receptor-mediated endocytosis, leading to a decrease in internalization.

As a final step, we wanted to determine whether TAs associate directly with InlB in a Gal-dependent manner. Towards this aim, we produced recombinant InlB from 1042 (**S9A Fig**), and assessed for any direct interaction with the *L*. *monocytogenes* cell wall. InlB adhered less to 1042Δ*gttA* than to 1042 and 1042Δ*gttA*::pPL2(*gttA*) (**Fig 6A**). Binding affinity quantification via surface plasmon resonance (SPR) showed that WTA polymers associate strongly with InlB when they possessed the Gal decoration (1042, 1042Δ*gttA* complement, 1033 complement) (**Fig 6B**). However, WTA polymers lacking Gal (1042Δ*gttA*, 1033) showed significantly reduced affinity, with a roughly 1000-fold decrease in K_A_. Similarly, LTA polymers associated with InlB in a Gal-dependent manner, however Gal had a less significant effect on this affinity, signified by the relatively high response of the species lacking Gal (**Fig 6C**). This suggests that both LTA and WTA polymers mediate InlB localization to the bacterial cell wall in a Gal-dependent manner, although the WTA in *L*. *monocytogenes* serovar 4b cells may play a more significant role. When expressed alone, the C-terminal Csa domain of InlB demonstrates a similar Gal-dependent TA affinity to the full-length protein, while InlB lacking Csa (InlBΔCsa) had negligible interaction (**S9C Fig**). As referenced previously, serovar 4c strains also harbor a Gal decoration on their WTA, although the position of the GlcNAc is at the Ribitol’s C2 position. Because this strain demonstrates an attenuated invasiveness, we wondered whether this was in part due to InlB surface expression. Interestingly, the 4c strain 1019 demonstrated a lower level of total cellular InlB and its purified WTA also showed a decreased level of association with InlB compared to the WTA of 1042 (**S10 Fig**). This suggests that the Gal-WTA structure of SV 4b is unique in its ability to strongly associate with InlB and retain it at the cell surface. Taken together, this establishes a clear link between a serovar-defining structure which is specifically targeted by bacteriophages, and the display and function of a canonical *L*. *monocytogenes* virulence factor (**see Fig 7**).

## Discussion

From our findings presented here, we can theorize a stimulating model, which suggests that 4b strains maintain their teichoic acids glycosylation pattern and ability to invade host cells at the cost of bacteriophage sensitivity. Actuated by phage predation, this model demonstrates several principles. First, it shows that bacteriophage can be used in the laboratory to target serovar-specific antigens, selecting for strains that convert to a different serovar. Second, it demonstrates that the 4b-specific WTA antigen may be a general contributor to virulence, as it significantly affects the strain’s ability to invade host cells. Third, it led to the discovery of a new mechanism: InlB, a canonical PrfA-dependent virulence factor of *L*. *monocytogenes*, requires galactosylated teichoic acids for proper surface localization in serovar 4b strains.

Bacteria can acquire resistance to phage infection through many means [44]. Adsorption of a bacteriophage to its host bacterium is the first step in the process of infection, and in the case of *Listeria*, phages face a wide diversity of surface polysaccharides that they can recognize. Since only a specific subset of *Listeria* harbor internal resistance mechanisms such as CRISPR-Cas [45,46], it is not surprising that the primary resistance mechanism appears to be via ligand modification, i.e. loss of WTA glycosylation [29]. The predator-prey relationship between *Listeria* and phages seems to have created a wide array of surface diversity, manifested by different WTA structures, and represented by serovar. Given that full WTA glycosylation renders the bacteria sensitive to phage attack, one can presume that a selective pressure must exist that causes the bacterium to maintain this pattern, despite the clear disadvantage. In fact, WTA glycosylation has been shown to confer certain other advantages, such as resistance to antimicrobial peptides [26], cold tolerance [47], and general virulence [24,48,49]. In other words, *Listeria* faces a classical trade-off situation: maintenance of fitness and virulence at the cost of bacteriophage sensitivity.

Bacteriophage A500 has a serovar 4b-specific host range [27]. However, temperate bacteriophages such as A500 are difficult to use as tools to study cell wall structure because of their propensity to form homo-immunity-inducing lysogens, meaning that they cannot exert a strong selective pressure on the bacterium under laboratory conditions that would ultimately lead to cell-wall-mediated resistance. In the laboratory, it is far more feasible to work with virulent phages for the purpose of creating BIMs through artificial selection, which our recently developed synthetic phage platform [37] allowed us to do. Predation by the (naturally) virulent phage A511 demonstrated that resistance to phage with a broad host range also involves loss of glycosylation, leading to a similarly attenuated invasion phenotype.

Challenge with the converted A500ΔLCR phage rapidly resulted in loss of galactosylated WTA via mutation of either *gttA* (encoding a galactosyltransferase) or *galE* (putative glucose-4-epimerase). *gttA* has 77% amino acid identity to *glcV*, which is thought to catalyze the addition of uridine diphosphate galactose (UDP-Gal) onto undecaprenol phosphate, an intermediate step before Gal addition to WTA in a 4c strain [24]. We hypothesize that *galE* produces UDP-Gal from UDP-Glc. Therefore, without this gene, Gal moieties are not available for addition to the WTA chain, but glucose moieties still are. UDP-Glc itself is made by a UDP-glucose pyrophosphorylase encoded by *galU* [50], which would explain why an inactivating mutation in *galU* leads to both a loss of Glc and Gal decorations from the WTA. Presumably, the same UDP-Gal is also used for addition onto the LTA chain, which is why 1042Δ*gttA* lacks Gal from the LTA chain as well.

The association of InlB with bacterial surface components, namely LTA, has been previously investigated [14,51]. However, the interaction of InlB with the cell wall is relatively weak, as heparin and purified LTA can induce the detachment of InlB [52]. While previous research has not yielded clear evidence for InlB association with WTA [15,52], our data clearly demonstrate a direct, Gal-dependent binding of InlB to both LTA and WTA; an association strong enough to mediate InlB’s surface display. Further, it was recently shown that InlB surface display in serovar 1/2 strains is somewhat dependent upon rhamnosylated WTA [25]. From the findings presented here, it is difficult to discern whether InlB relies more heavily on Gal-WTA or Gal-LTA, since manipulation of Gal decoration of one TA type only was not possible. However, upon scrutiny of the estimated binding affinity (K_A_), InlB still associated well with LTA lacking Gal, whereas its interaction with WTA dropped drastically. These observations allow us to hypothesize that InlB relies more heavily on WTA over LTA for its localization. If indeed a complete TA glycosylation pattern is necessary for high level InlB localization, one can theorize that serovar 1/2 and serovar 4b strains have a tendency to be more virulent (they do cause the vast majority of infections), in part because they possess more glycosylated TAs relative to other serovars. Heterologous expression of intact *gttA* in the serovar 4d strain WSLC1033 promoted correct cell wall localization of InlB, resulting in an increased level of invasiveness, suggesting that this 4d strain (and likely others) may have evolved from a 4b following phage predation. Some data exist already in other serovars that complement this finding, although things become more complex as one moves beyond the 4b-4d model presented here. In one study, PrfA activation in the serovar 4a strain M7 led to an overexpression of virulence factors, but the majority of InlB was released into the medium [53], indicating that serovar 4a teichoic acids are not sufficient to retain InlB. Serovar 4c on the other hand does harbor a Gal decoration in its WTA, although at the ribitol C2 position instead of C4 like in serovar 4b. As InlB binds both gal-LTA and WTA, demonstrating a lack of structural specificity, it is curious that the WTA from 4c does not seem to associate with InlB despite its Gal decoration. The lack of invasiveness of the 4c strain therefore likely derives from a similar reason as seen in 4d, and suggests that the Csa domain actually does have some structural preference.

The striking invasion phenotype observed in HeLa cells, which are devoid of E-cadherin, fits well with our observation of drastically lowered cell-wall-associated InlB in mutants lacking galactosylated TAs. Similarly, we perceived a stronger invasion attenuation in HepG2 cells relative to Caco-2, in agreement with InlB being a major mediator of invasion in hepatocytes[16,35,54], with cMet functioning as the primary receptor for activation of the invasion machinery [36]. Our data rules out that InlA-mediated invasion is affected upon loss of Gal and strongly suggests that InlB is the primary mediator of the observed invasion phenotype. However, we cannot state with absolute certainty that InlB is the sole actor, especially given that a few other examples of surface-associated proteins are known to play a minor role in invasion. The fact that our LRC data demonstrated a few other candidate receptors preferably interacting with the 4b cell wall suggests that other pathways not related to cellular invasion may be affected.

The well-established importance of InlB for invasion of the liver, spleen and placenta [54,55], suggests a virulence implication, although it is understood to be mild. 1042Δ*gttA* showed a strong virulence attenuation *in vivo*, significantly more than what was observed in a 1042Δ*inlB* mutant. However, similar to what has been previously suggested [24], actin tail formation appears to also be affected in this Gal-deficient mutant, likely due to ActA dysfunction. This implies that Gal-TAs have a relationship with a second *Lm* virulence factor, although not one that involves direct adherence, since ActA does not possess a Csa domain. Since InlB is known to show a much greater virulence effect in an *actA* background [56], we can hypothesize that the 1042Δ*gttA* virulence attenuation is due to loss of function of both the InlB and ActA virulence factors. Of course, further evaluation will be needed to understand how Gal-TAs affect the function of ActA.

It has been argued that the use of antibacterials that target virulence factors and not genes essential for bacterial survival, would greatly decrease rates of antibiotic resistance due to the lack of selective pressure for survival [57]. Although targeting of virulence factors is less likely to work in established infections, it could serve a purpose in more preventative measures, especially against antibiotic-resistant pathogens, and those with specific factors necessary for survival and proliferation in a host. The cell wall of *Listeria* and the proteins associated with it, are responsible for most interaction with the mammalian host, and for serovar determination, meaning that many of its structures present potential drug targets [58]. We believe that here we have identified one such novel factor that can be targeted by bacteriophage. Future research will undoubtedly focus on other changes in the bacterium that result from phage predation that led to attenuated viability in the host and environment, and whether the TA-mediated invasion mechanism present here applies more broadly, as such evidence would further support bacteriophages as potential anti-virulence agents in other Gram-positive pathogens.

## Materials and methods

### Bacterial strains, plasmids, phages and growth conditions

All bacterial strains, plasmids and phages used in this study are listed in S1 Table. *E*. *coli* XL1-Blue (Stratagene) used for cloning and plasmid construction was routinely cultured in Luria–Bertani (LB) broth at 37°C. The *E*. *coli* BL21-Gold strain was used for protein expression. *L*. *monocytogenes* strains were grown in 1/2 brain heart infusion (BHI), at 30°C with shaking when working with phages, or at 37°C when the cells were used for infection studies. *L*. *monocytogenes* mutants were constructed in a WSLC1042 background [59]. Genomes of other serogroup 4 strains are referenced in Sumrall et al., 2016 [60]. For the selection of plasmids, growth medium was supplemented with antibiotics as indicated: ampicillin (100 μg ml^−1^ for *E*. *coli*), chloramphenicol (10 μg ml^−1^ for *E*. *coli* and *L*. *monocytogenes*), erythromycin (300 μg ml^−1^ for *E*. *coli*, 10 μg ml^−1^ for *L*. *monocytogenes*). Propagation of bacteriophages A511, A500, and A500ΔLCR was performed using *L*. *monocytogenes* strain WSLC1042, and purification was performed as previously described [61,62]. Phage stocks were stored at 4°C in CsCl and diluted into SM Buffer (100 mM NaCl, 8 mM MgSO_4_, 50 mM Tris-HCl, pH 7.5) before use. Host strains were grown overnight in BHI broth for adsorption and infection assays. To determine minimal inhibitory concentrations to cefotaxime, serial dilutions of overnight cultures were prepared in a 96-well plate format [63]. For growth curve determination, overnight cultures were diluted to an OD of 0.01 in triplicate in a 96-well plate, and the OD_600_ was measured every hour for 14 hours on a plate reader set to 37°C.

### Construction of *L*. *monocytogenes* knockout and complementation strains

Deletion knockouts were produced via allelic exchange. 500bp flanking regions of the gene in question from WSLC 1042 were engineered as strings and ligated into the thermosensitive vector pAUL-A or pHoss1, followed by transformation into *E*. *coli* XL1-Blue. The primers 5’-GTAAAACGACGGCCAGT-3’ and 5’-GCTATGACCATGATTACGC-3’ (for pAUL-A); and 5’-GGATTTACTCCTGGAGCTGGT-3’ and 5’-CGGAAGCGAGAAGAATCATAATGG-3’ (for pHoss1) were used to confirm and sequence vector inserts. Complemented strains were produced using the integrational vector pPL2 [64]. The desired gene containing roughly 100bp of of upstream sequence was amplified by PCR and ligated into the EcoRI and BamHI restriction sites. The single site integration vectors were introduced into either WSLC 1042 or WSLC 1033 and the genes expressed from their native promoter. GFP and RFP expressing strains were also constructed using the pPL3 and pPL2 integration vectors, respectively [64,65]. Insert sequences were confirmed using primers F: 5’-TCACTAAAGGGAACAAAAGCTG-3’ and R: 5’-GACGTCAATACGACTCACTATAGG-3’.

### Isolation of bacteriophage insensitive mutants (BIMs) and adsorption analysis

To isolate bacteriophage insensitive mutants, log phase *Listeria* cultures were diluted 100-fold in 5 mL 1/2 BHI medium. Bacteriophages A511 or A500ΔLCR were added at a 10:1 PFU:CFU ratio and the bacteriophage/cell mixture incubated overnight. Initially, the cultures reduce in turbidity due to phage lysis, followed later by regrowth. The procedure was repeated twice, each time using the culture with the surviving cells from the day before. Several colonies were picked from this final culture and evaluated for the ability of the original phage to adsorb via a phage pulldown assay, which was performed as previously described [27]. The number of phage adsorbed to the bacterial surface was evaluated by plaque assay and expressed as the relative adsorption to the mutant relative to the WT. Genomes of select mutants demonstrating significant decrease in phage adsorption were re-sequenced via Illumina to detect any genetic changes (see below).

### Phage lysis curves

To evaluate phage lysis kinetics of phage A500ΔLCR, an overnight culture of WSLC 1042 was diluted to OD_600_ of 1, 0.1 and 0.01 in 1/2 BHI supplemented with 1 mM CaCl_2_. 10 μL of a 10^9^ PFU/mL bacteriophage stock was prepared in wells of a 96-well plate and 200 μL diluted overnight stocks were added. Bacterial growth was monitored at 30°C by measuring OD_600_ values over 22 hours on a plate reader in triplicate.

### Production and verification of the virulent bacteriophage A500ΔLCR

Phage rebooting was performed using a protocol adapted from Kilcher et al., 2018 [37]. 15 mL of WT phage A500 lysate at ~10^9^ PFU/mL was filtered through a 0.2 μm. 10 μL RNase A (10 mg/mL) and DNase I (1 U/μL) were added to approximately 15 mL lysate and incubated for 2 hours at room temperature. Precipitation solution (3 M NaCl, polyethylene glycol 8000) was added in a ratio of 1:2 (precipitation solution/lysate), mixed by inversion and incubated for 1–2 days at 4°C. The precipitated phage lysate was centrifuged (10,000 x g, 10 min, 4°C), the pellet was resuspended in 500 μL SM buffer and transferred to a fresh tube. Unsolubilized PEG was removed by spinning for ten seconds at maximum speed in a tabletop centrifuge. 10 μL EDTA (0.5 M, pH 8) and proteinase K (20 mg/mL stock solution) were added to the phage solution and the mixture incubated for 30 minutes at 50°C. 600 μL binding buffer from the High Pure Viral Nucleic Acid Kit (Roche) was added and loaded onto the provided columns. The quality of the genomic DNA was assessed by running 50 ng on a 1.1% agarose gel using pulsed-field gel electrophoresis in TBE buffer. The sample was run for 5 hours at a temperature of 14°C, with an initial switch time of 0.6 seconds and a final switch time of 1.6 seconds, at a dielectric strength of 8 V/cm and an angle of 120°.

The A500 bacteriophage genome was partitioned (*in silico*) into four DNA fragments of approximately equal size that feature 40 nt overlaps with neighboring fragments. The partitioned genome was constructed to lack the genes *gp32*, *gp33* and the integrase (e.g. lysogeny control region or LCR, nucleotide region 24281 to 25852). The phage genome fragments were amplified from purified phage DNA by PCR using Phusion DNA polymerase. Synthetic genomes were assembled with the Gibson method for 1 h at 50°C. For the assembly reaction, 0.03 pmol of each DNA fragment was used in a 15 μL reaction. For transfection of L-form cells and subsequent rebooting, 20 μL of the assembly reaction was used as input DNA.

Small volumes (4–5 μL) of a Rev2L culture were inoculated into 1 mL prewarmed DM3 growth medium supplemented with PenG (200 μg/mL), suspended using a vortex mixer at low speed, and incubated without agitation at 32°C for 96 hours. The resulting L-form culture was suspended by pipetting and adjusted to an OD_600_ of 0.15 using DM3 medium. 100 μL OD-adjusted Rev2L culture was mixed with 10–20 μL phage DNA (genomic or synthetic) in a 50 mL Falcon tube. 150 μL sterile 40% PEG 8000 solution was added and mixed thoroughly by pipetting. After 5 minutes of incubation, 10 mL pre-warmed DM3 medium (30°C) was added and mixed and the transfection reaction was incubated without agitation at 32°C for 24 hours. The L-form transfection reaction was assayed for matured phage particles using the soft-agar overlay method, using strain WSLC 1042. To confirm the for loss of the LCR within the A500 genome, this genomic region was amplified by PCR amplified and sequenced. To test for virulent properties, WT A500 and A500ΔLCR were each used to infect liquid cultures of strain WSLC 1042. For the cultures infected with phage A500 or A500ΔLCR phage integration was verified by PCR using primer sets A500_int or A500_intsite which anneal either to the A500 genome itself or to the 1042 genomic region adjacent to the integration site, respectively.

To test whether A500ΔLCR possesses a virulent lifestyle, an overnight culture of WSLC 1042 was grown and diluted to OD_600_ of 1, 0.1 and 0.01 in 1/2 BHI supplemented with 1 mM CaCl_2_. 10 μL of a 10^9^ PFU/mL bacteriophage stock was prepared in wells of a 96-well plate and 200 μL diluted overnight bacterial cultures were added. Bacterial growth was monitored at 30°C by measuring OD600 over 22 hours on a plate reader.

### Illumina re-sequencing of bacteriophage resistant mutants

*L*. *monocytogenes* genomic DNA was extracted using a bacterial genomic DNA isolation kit (Sigma-Aldrich). Lysis of the bacteria was done using the laboratory produced Ply511 endolysin. Library preparation and sequencing were performed by the Functional Genomics Center Zurich (University of Zurich, Switzerland). The library was prepared using Illumina TruSeq Nano DNA Library reagents, and sequenced at 2 x 250bp paired-end reads for 500 cycles. The CLC genomics workbench software package (Qiagen) was used to map the reads to the WSLC 1042 reference genome sequence, identifying high-confidence changes in the genome of the BIM strains.

### Serotyping

Serotyping procedures were performed using the traditional method, as described by Seeliger & Höhne, 1979 [66].

### Protein purification

Expression and purification of His-tagged *Listeria* Internalin B and its variants were carried out as previously described [67]. Briefly, full length *inlB*, a truncated version lacking the sequence coding for the the C-terminal GW domains (*csa*, the first 398 AAs of the protein), or a version containing only the sequence coding for the GW domains (last 233 AAs of the protein) were amplified by PCR and ligated into the expression vector pET302 NT-His and transformed into *E*. *coli* strain BL21-gold. InlB and the two truncated variants were purified from 1- liter culture using a nickel-affinity column and the purity of the protein was evaluated on an SDS-PAGE gel, as previously described [68].

### Cell culture techniques

The Caco-2, HepG2, GPC16 and HeLa cell lines used for *in vitro* assays were cultured at 37°C with 5% CO_2_ in DMEM GlutaMAX (Gibco), supplemented with sodium pyruvate, 1% non-essential amino acids and 10% FBS. For both the invasion and adherence assays, the human cells were diluted to a concentration of 2-4x10^5^ cells/mL and seeded onto 96-well plates (24-well plates for GPC16) in triplicate the day before performing the experiment (for GPC16, 2 days before). On the day of infection, bacteria were grown at 37°C to an OD_600_ of 0.8–1.0, before being washed twice in PBS and diluted in DMEM lacking FBS to OD_600_ 0.01, which corresponds to a multiplicity of infection (MOI) of ~100. Human cells were washed twice with DPBS and bacterial suspensions (200 μL per well) were added to the human cells (1 mL for GPC16). For the adherence assay, bacteria were allowed to settle onto the seeded monolayer for 10 min, after which the excess was washed away three times with 200 μL DPBS. For the cellular invasion assays, bacteria were added in the same manner, and incubated for 2 h at 37°C. Cells were washed twice with DPBS and incubated a further 1 h in normal growth medium (with FBS) supplemented with 40 μg/mL gentamicin to kill any remaining extracellular bacteria, followed by another two rounds of washing with DPBS. For both invasion and adherence assays, mammalian cells were lysed with 0.5% Triton-X-100, serially diluted, and 10 μL were spot-plated onto BHI agar plates. Experiments involving CD97, THR and Gc1qR inhibition were performed as stated above evaluating only 1042 WT invasion following treatment of HeLa cells with an anti-CD97 monoclonal antibody at a 1:200 dilution for 30 min in infection medium (ab108368: Abcam), a thyroid hormone receptor antagonist at 5 μM for 60 min in infection medium (1-850-CAS 251310-57-3: Calbiochem) or an anti-Gc1qR monoclonal antibody at a 1:200 dilution for 30 min in infection medium (ab24733: Abcam). The number of bacteria that had adhered or invaded was expressed as a fraction relative to the invasion rate of strain WSLC 1042, which was arbitrarily set to 1 for each replicate. For all invasion or adhesion experiments, at least three biological replicates were performed, each time with three technical replicates.

### Fluorescence microscopy analysis

HeLa or Caco-2 cells were seeded onto 2-well ibiTreat 8-well chamber slides (Ibidi) at a concentration of 4x10^5^ cells/mL. *Listeria* strains expressing either GFP or RFP were used to perform invasion assays, which were performed as described above. Following infection, cells were fixed in 4% formaldehyde, treated for 15 minutes with 50mM NH_4_Cl to quench autofluorescence, permeated with 0.1% Triton X-100 for 2 min and stained with 0.1 μg/ml Phalloidin-TRITC and 10 μg/ml Hoechst DNA stain (Sigma) diluted in PBS. Microscopy analysis was performed on a Leica TCS SPE confocal system (Leica Microsystems GmbH, Germany) equipped with a HCX PL FLUOTAR 100.0 × 1.30 oil objective. The images were analyzed and prepared for publication using the Leica application suite software. Quantification of the invasion rate was performed by counting the intracellular bacteria visible within the entire infected cell. One hundred cells from three separate chamber slides were counted altogether. The percentage of infected Caco-2 cells was determined by counting one hundred total cells from three separate chamber slides from randomly chosen areas of each slide, and determining the number which contained intracellular fluorescent bacteria. To evaluate extracellular killing efficacy with the gentamicin treatment, a 5-minute infection was performed, followed immediately by washing and gentamicin treatment (as in infection assays), and the resulting CFU evaluated, which yielded zero colonies. To evaluate actin tail formation, the same procedure was followed, except that cells were infected for a total of six hours to allow for full formation of actin tails. Visualization was done in an identical manner, as described above. For InlB surface staining of *L*. *monocytogenes*, cells were grown to mid-log phase, washed with PBS and treated with 100 μg/ml lysozyme for 30 min to make InlB more accessible for immunostaining and blocked in 1% BSA/PBS. Cells were further washed and stained with a custom-produced InlB polyclonal antibody at a 1:100 concentration (Invitrogen) for 30 min, washed again in PBS and further incubated in Alexa Fluor 594-conjugated goat anti-rabbit secondary antibody. After a final wash step, cell suspensions were applied to a glass slide and imaged as described above.

### Evaluation of direct adherence between WTA and host cells

Investigation of whether WTA directly adheres to Caco-2 cells was performed as previously described [39,69] with some modifications. Briefly, for the competitive inhibition assay, Caco-2 cells were seeded in 96-well plates in triplicate, as stated above. Before addition of bacteria, cells were incubated with 500 μg/mL purified WTA polymer isolated from strains 1042 WT or 1042Δ*gttA*. Bacteria were prepared, added and evaluated in the same manner as in the standard adherence assay described above.

For the bead assay, a phenol colorimetric assay was used to confirm WTA polymer binding to the amine-coupled fluorescent beads. Beads coated with WTA were added to seeded Caco-2 cells at a ratio of 400:1 beads to cells. Adherence of the beads to Caco-2 cells was evaluated in a plate reader by measuring the total amount of fluorescence emitted from the plate, and expressed as relative fluorescent units.

### Quantitative RT-PCR

Five milliliters of each culture at logarithmic phase was centrifuged and total RNA was extracted using the PureLink RNA Mini Kit (Ambion Inc), following the manufacturer’s protocol. DNA was removed using DNAse I (ThermoFischer Scientific). The resulting RNA concentration was analyzed spectrophotometrically, and reverse transcribed using the TaqMan reverse transcription reagents kit (Applied Biosystems), using random hexamers. qPCR was performed using SYBR Select Master Mix (Applied Biosystems) on a Rotor-Gene 6000 (Corbett Life Science) device with the following cycling conditions: 50°C for 2 min, 95°C for 10 min, 30 x (95°C for 15 s, 60°C for 2 min). The primers used for measuring the *inlB* transcript levels were 5’-GAGTAACCCAACCACTGAAAGA-3’ (forward) and 5’-CGGAGGTTTAGGTGCAGTTAT-3’ (reverse). 16s rRNA primers was used as normalization controls: 5’-CCGTATTGTAGTCGCTGATTCT-3’ (fwd) and 5’-CAAAGTTGCTTCTTCGTCGATTT-3’ (rev). The experiment was performed in three biological replicates and expressed as the mean of the fold change of *inlB* expression from all three experiments.

### Western blot analysis

Cell fractionation experiments were performed as previously described [14], with minor modifications: the endolysin Ply511 was used to digest the cell wall, leading to protoplast formation in an osmotically balanced sucrose medium. To verify proper fractionation, western blot was performed with supernatant, cell wall, membrane and cytoplasmic fractions. Surface protein extracts were prepared as previously described [70].

Protein extract concentrations were measured using the BCA Protein assay kit (Pierce Biotechnology), and 5μg of sample was mixed with SDS sample buffer containing 0.5% β-mercaptoethanol (in a total sample volume of 40 μL), boiled for 5 min, and run on a Pre-cast Any kD Mini-PROTEAN TGX gel. All proteins were transferred onto PVDF membranes using an iBlot dry blotting system (Invitrogen), followed by incubation in SuperSignal western blot signal enhancer (only for InlB detection, Pierce Biotechnology). Immunodetection was performed with an iBand flex western device (Invitrogen), according to the manufacturer’s protocol. Immunodetection with the InlB antibody was done at a concentration of 1:400, InlA (Biorbyt) at a concentration of 1:2000 For the detection of LTA, whole cell extracts were prepared of the *Listeria* strains and as previously described [12,71]. For western-blot analysis, a polyglycerol-phosphate-specific LTA antibody (Clone 55 from Hycult biotechnology) and an HRP-conjugated anti-mouse IgG (Cell Signaling Technologies, USA) were used.

### Surface plasmon resonance analysis

Sensorgrams of representing binding of purified *Listeria* WTA or LTA polymers to various immobilized internalin B proteins variants were obtained using surface plasmon resonance (Biacore X, GE healthcare, Glattbrugg, Switzerland) as previously described [8] with slight modifications. Briefly, the carboxymethylated surface of a CMD500L chip (Xantec bioanalytics GmbH, Duesseldorf, Germany) was coated with 0.25 mg/mL internalin B or truncated variants at a flow rate of 5 μL/min; 10mM sodium acetate pH 4.0, using the amine coupling procedure according to the manufacturer’s manual. For specificity studies, 7 μM of purified WTA or LTA samples (analyte) isolated from different *Listeria* strains were flowed through both cells in running buffer (10 mM Bis-Tris, 100 mM NaCl, 3.4 mM EDTA, 0.005% Tween 20, pH 6) at 10 μL/min at 25°C. For each analyte, association was measured for 180 s and dissociation was measured for 720 s. After each injection, the surface was regenerated by injecting regeneration buffer (1 M NaCl, 50 mM Tris, pH 9) for 45s, at a flow rate of 10 μL/min. This cycle was repeated after each measurement. For kinetic studies, the interaction between InlB and WTA/LTA used at concentrations ranging from 0 to 7 μM was determined again using a flow rate of 10 μL/min at 25°C. For each concentration, the association was measured for 180 s, and the dissociation was monitored for 720 s. The surface was then regenerated with regeneration buffer prior to the next measurement. For all curves, the ‘two-state confirmation change’ model gave the best fit and was used for calculation of K_A_ (the inverse of K_D_) values.

### Binding of purified InlB to different *L*. *monocytogenes* strains

Aliquots of overnight cultures of *L*. *monocytogenes* strains were washed with PBS and the concentration was adjusted to an OD_600_ of 1.0 and then treated for 30 minutes with 10 μg/mL of lysozyme. 125 μL of washed cell suspension was mixed with 25 μL of 20 μM purified InlB and incubated at RT for an additional 30 minutes, following which the bacterial cells were washed twice with PBS and the pellet resuspended in sample buffer. SDS-PAGE gel electrophoresis followed by western blot was then performed using a 6x-His Tag monoclonal antibody (MA1-21315-HRP, Thermo Fischer Scientific) to quantify the overall amount of exogenous, tagged InlB that bound to the bacterial cell wall.

### WTA extraction and structural analysis

WTA was purified from the indicated *L*. *monocytogenes* strains as previously described (Eugster & Loessner, 2011). Purified WTA polymers were depolymerized into monomeric repeating units by hydrolysis of the phosphodiester bonds using 48% hydrofluoric acid for 20 h at 0°C. The degraded products were further purified using a Superdex 200 size exclusion column (GE healthcare, Glattbrugg, Switzerland) in distilled water at 25°C (flow rate, 0.4 mL/min). Fractions containing material smaller than 1 kDa were identified using a UV detector at 205 nm, pooled and dialyzed (MWCO 100–500 Da, Spectra laboratories, Inc.) against distilled water. The WTA monomers were then lyophilized and subjected to UPLC-MS/MS for compositional and structural analysis, as previously described [8].

### LTA isolation and NMR analysis

For the isolation of LTA from *L*. *monocytogenes*, the strains were grown overnight in 2 L BHI medium and the bacteria were harvested by centrifugation. LTA was purified and then analyzed using one dimensional (1D) ^1^H nuclear magnetic resonance (NMR) as described previously [15,72]. Briefly, LTA was butanol-purified and isolated from the remaining cellular extracts by hydrophobic interaction chromatography using a 24 x 1.6 cm octylsepharose column. Fractions containing LTA were identified by western blotting using a polyglycerolphosphate-specific antibody (Clone 55 from Hycult biotechnology), pooled, extensively dialyzed against water and subsequently lyophilized. 2 mg LTA were suspended and lyophilized twice in 500 μl D_2_O of 99.96% purity. In the final step, the LTA extracts were suspended in 500 μl D_2_O of 99.99% purity and NMR spectra were recorded on a 600-MHz Bruker Advance III spectrometer equipped with a TCl cryoprobe. To ensure accurate integration of the signals, NMR spectra were recorded at 303 K with a total recycling time of 5 s and a ^1^H flip angle of approximately 30°. Two independent LTA extractions were performed for each strain. The data were analyzed using the Topspin 3.5 software (Bruker Biospin, Ltd) and the spectra annotated according to previously published NMR spectra [15,73–75]. Western blots to detect glycerol phosphate were performed also as previously described using whole-cell extracts.

### HATRIC ligand receptor capture

HATRIC-LRC was performed as previously described with small modifications. Briefly, 10^9^ bacterial cells were coupled to 50 μg HATRIC for 1.5 h with slow rotation at 22°C in 100 μl 25mM HEPES (pH 8.2). Adherent HeLa cells were mildly oxidized with 1.5 mM NaIO_4_ for 15 min at 4°C in the dark. Bacteria-HATRIC conjugates were re-suspended in PBS pH 7.4 containing 5 mM 5-methoxyanthranilic acid (5-MA) and added to the cells (MOI 100:1). Cells were incubated for 30 min at RT. Upon receptor capture, cells were collected by gentle scraping and pelleted. Cell pellets were processed as previously described [43].

### Intravenous mouse infection

Animal procedure used in this study is in agreement with the guidelines of the European Commission for the handling of laboratory animals, directive 86/609/EEC. It was approved by the ethical committees CETEA/CEEA n°89 under the number "2015–0015" and by the Ministry of Higher Education, Research and Innovation under the number APAFIS#9754–201704261739288 v1. L. monocytogenes overnight cultures were diluted in Brain-Heart-Infusion (BHI) medium to reach mid-log growth phase. 200 μl of bacteria in PBS (5x10^3^ CFUs/animal) were injected in the vein tail of C57BL/6J mice. To monitor bacterial burden, organs were removed from killed animals 3 days post infection and homogenized in PBS. Serial dilutions of tissue suspensions were plated on BHI agar plates. After 24 h of incubation at 37±1°C, colony-forming units were counted.

### LC-MS/MS analysis

Tryptic peptide fractions were reconstituted in 20 μL 5% acetonitrile/0.1% FA/doubly distilled water, and 2 μg per sample was loaded onto an EASY nano-HPLC system (Proxeon) equipped with a RP-HPLC column (75 μm x 10.5 cm) packed in-house with 10 cm stationary phase (Magic C18 AQ 1.9 μm, 200 Å, Michrom BioResources). The HPLC was coupled to a QExactive plus MS (Thermo Scientific) equipped with a nano-electrospray ion source (Thermo Scientific). Peptides were loaded onto the column with buffer A (0.1% FA) and were eluted with buffer B (99.9% ACN, 0.1% FA) at a flow rate of 300 nL/min. The MS was operated in data-dependent manner, with an automatic switch between MS to MS/MS scans. High-resolution MS scans were acquired (70000, target value 106) within 300 to 1700 m/z. The 20 most intense precursor ions were fragmented using higher-energy collisional dissociation (HCD) to acquire MS/MS. Unassigned and singly charged ions were excluded from HCD, and dynamic exclusion was set to 15 s. RAW data were converted to mzML using MSconvert. Fragment ion spectra were searched with COMET (v27.0) against UniprotKB (v57.15, Homo sapiens) containing common contaminants. The precursor mass tolerance was set to 20 ppm. Carbamidomethylation was set as a fixed modification for cysteine and oxidation of methionine as a variable modification. Probability scoring was done with PeptideProphet and ProteinProphet of the Trans-Proteomic Pipeline (v4.6.2). Protein identifications were filtered for a FDR of ≤1%. For label-free quantification, proteins were filtered for membrane-associated proteins and non-conflicting peptide feature intensities extracted with Progenesis QI (Nonlinear Dynamics). Protein fold changes and their statistical significance between paired conditions were tested using at least two fully tryptic peptides per protein with SafeQuant (v2.3.1). Proteins were considered candidates if they showed a fold-change of 4 or higher and an adjusted p-value of 0.01 or lower.

### Statistics

All experiments were performed with n equal to or greater than 3 unless otherwise stated. For all invasion and adherence assays, comparisons between mutant and WT, and WT and complementation strains were evaluated for statistical significance using an unpaired t-test, unless in the cases where more than two groups were compared, where one-way ANOVA comparisons were performed, as indicated in the figure legends. Mann-Whitney tests were performed on mouse CFU counts to determine statistical significance.

## Supporting information

S1 Fig MS chromatographic comparison of three serovar 4b strains and 1042_A511BIM. **(A)** The resulting WTA monomer structures of the indicated strains, as determined by UPLC-MS/MS (data representative of two separate extractions) and the corresponding serovar designation determined via a slide agglutination test. On the chromatograms, peaks are labeled with their corresponding retention time and *m/z*. **(B)** The resulting WTA monomer structures of the indicated strains, as determined by UPLC-MS/MS (data representative of two separate extractions) and the corresponding serovar designation determined via a slide agglutination test. On the chromatograms, relevant peaks are labeled with their assigned structures based on the *m/z*. The dominant peak at 1 min appearing in the *galU* complemented strain represents the ionized species that elutes without separation, resulting from incomplete de-polymerization.
(TIF)

S2 Fig Construction of the virulent A500ΔLCR phage by rebooting, and the resulting consequences. (**A**) Schematic of the phage A500 genome. Highlighted are the three genes (integrase, gp32, gp33) made up of 3191 bp, which were deleted to produce the mutant phage A500ΔLCR. (**B**) PCR detection of the A500 genome within the designated phage integration region of 1042 infected with either A500 phage (WT), or A500ΔLCR (KO). The presence of a band corresponds to a positive result for integration i.e. lysogen formation. (**C**) Growth curves over a 12-hour period of 1042 WT challenged with the indicated MOI of phage A500ΔLCR. (**D**) Table representing the three BIMs (far left column) discussed in the text, the mutations identified via Illumina re-sequencing, the gene that the mutations fall in, and the resulting phenotype/serotype change. **(E)** Liquid chromatographic separation and MS-based identification of WTA monomer residues from a select BIM in a 1042 background (harboring a mutation in a gene encoding a UDP-Glucose-epimerase). **(F)** Phage affinity evaluation using the WT phage A500 against the indicated *L*. *monocytogenes* strains, as determined by phage pulldown assays (means normalized to 1042 WT ± SEM, n = 3 for all samples; ****P < 0.0001; ns, not significant relative to 1042 WT, as determined via a one-way ANOVA using 1042 WT as a reference).
(TIF)

S3 Fig Evaluation of TA deletion mutants from a WSLC 1042 background. **(A)** Liquid chromatographic separation and MS-based identification of WTA monomer residues from 1042 and the indicated mutants. The peaks for 1042 WT are labeled with their assigned structures based on the *m/z*. The chromatograms are aligned on the same time axis to allow for proper comparison. (data is representative of two separate extractions). The dominant peak at 1 min appearing in the 1042Δ*gttA* mutant strain represents the ionized species that elutes without separation, resulting from incomplete depolymerization. **(B)** Growth curves (measuring OD600) of the indicated mutants measured over the course of 14 hours (each data point represents n = 3 measurements, error bars were eliminated for visual clarity). **(C)** Estimated cefotaxime MIC for the indicated mutants (for all, n = 3).
(TIF)

S4 Fig Structural determination of LTA monomers via WB and NMR. **(A)** Upper panel: Relative LTA decoration detection as determined by western blot of whole cell extracts using an antibody recognizing undecorated glycerol phosphate (representative of n = 3 blots). Positive signal represents undecorated LTA. Lower panel: Coomassie stain of the same blot to demonstrate equal sample loading. **(B)** NMR spectra of the repeating units of LTA from the indicated strains. Labeled peaks represent the major protons in the sample, while galactosylated protons are highlighted in yellow. The assigned structures for each strain are indicated on the right. The unlabeled major peaks are derived from residual citrate buffer used during the extraction process.
(TIF)

S5 Fig Microscopic evaluation of 1042Δ*gttA* invasion and actin tail formation within Caco-2 cells. **(A)** Fluorescence microscopy of 1042-GFP infecting a Caco-2 cell monolayer stained with Phalloidin-TRITC and Hoechst (image is representative of three individual experiments; contrast adjusted for clarity). **(B)** Percent of Caco-2 cells containing intracellular 1042-GFP or 1042Δ*gttA*-GFP, as determined via direct observation using fluorescence microscopy as in (A) (numbers are summed from counting fifty cells in two individual experiments). **(C)** The number of intracellular *L*. *monocytogenes* per Caco-2 cell, as determined by fluorescence microscopy as in (A) (each dot represents a single Caco-2 cell, and bars signify the mean intracellular *Lmo* per cell; numbers were determined by counting fifty cells from two individual experiments; significance was determined by comparing means). Extracellular *Listeria* were killed and eliminated by gentamicin treatment followed by vigorous washing. **(D)** Actin tail formation in Caco-2 cells six hours following infection by the indicated strains expressing GFP. Actin stained by phalloidin-TRITC.
(TIF)

S6 Fig MS-based WTA analysis following heterologous *gttA* expression in strain 1033. Liquid chromatographic separation and MS-based identification of WTA monomer residues from the indicated strains (left). Relevant peaks are labeled with their assigned structure. The chromatograms are aligned on the same time axis to allow for proper comparison (data is representative of two separate experiments). Predicted WTA monomer structures with the corresponding serovar designation, as determined via a slide agglutination test.
(TIF)

S7 Fig Evaluation of direct WTA-Caco-2 cell adherence. **(A)** Relative adherence of 1042Δ*gttA* and 1042Δ*gttA*::pPL2(*gttA*) complement compared to 1042 WT, as performed by a 10-minute infection assay in Caco-2 cells (left) and HepG2 cells (right) (means normalized to 1042 WT ± SEM; for both, n = 3; *P<0.05; **P<0.01; ns, not significant). **(B)** Caco-2 cell adherence of 1042 *L*. *monocytogenes* cells incubated in 500 μg/mL of WTA from 1042 or 1042Δ*gttA* relative to cells incubated in PBS (control) (mean ± SEM; n = 3; *P<0.05; ns = not significant). **(C)** Adherence of amine-coupled fluorescent latex beads coated with purified WTA from 1042, 1042Δ*gttA* or uncoated, determined by measuring total fluorescence on a 96-well plate and expressed as arbitrary fluorescence units (ns = not significant compared to uncoated beads control; mean ± SD; n = 8).
(TIF)

S8 Fig HATRIC-LRC identifies target receptors of recombinant InlB (rInlB) on the surface of HeLa cells. **(A)** Transferrin (TRFE) was used as a positive control. Results are presented in a volcano plot where relative fold changes of proteins (Log_2_ scale) are plotted against their respective log-transformed, false-discovery rate (FDR)-adjusted values enabling quick visual identification of proteins that display statistically significant changes. Target receptors are defined as proteins with a fold change greater than 4 and p-value equal to or smaller than 0.01, corresponding to the white space of the plot. Known receptors are shown in red and proteins originating from the ligand are marked in green (data is representative of three individual experiments). **(B)** Relative invasiveness of 1042 WT determined by infecting HeLa cells for three hours following treatment with a Thyroid hormone receptor antagonist (THR inhibitor.), an anti-CD97 monoclonal antibody, or an anti-gC1qR monoclonal antibody (invasion rate normalized to untreated control ± SEM; n = 3. *P < 0.05; ns, not significant relative to untreated control, as determined by a one-way ANOVA).
(TIF)

S9 Fig Association between WTA/LTA polymers and rInlB. **(A)** SDS-PAGE analysis of purified N-terminally His-tagged full-length InlB (71.25 kDa), InlBΔCsa (InlB lacking the C-terminal Csa domain containing the GW repeats, 44.78 kDa) and the Csa domain alone (26.48 kDa). **(B)** TA dose response example titrations of 1042 WTA/LTA (RU: relative units), against immobilized InlB (data used for estimated K_A_ values in Fig 5). **(C)** Binding kinetics of immobilized Csa (upper) or InlBΔCsa (lower) with WTA (left) or LTA (right) polymers extracted from the indicated strains, as determined by surface plasmon resonance analysis (RU: relative units; data is representative of two individual experiments, each time using newly purified constructs and WTA/LTA extracts).
(TIF)

S10 Fig Evaluation of total InlB expression and interaction with WTAs in the serovar 4c strain 1019. **(A)** Western blot of total *Lmo* extracts from the stains 1042, 1042Δ*gttA* and 1019 (which harbors galactosylated WTA, but at a different orientation from 4b), detected using an anti-InlB antibody. A ponceau stain of the entire nitrocellulose membrane before blotting to demonstrate equal loading is shown in the lower panel. **(B)** Binding kinetics of immobilized Csa from 1042 with WTA polymers extracted from the indicated strains (compare to S9C), as determined by surface plasmon resonance (RU: relative units).
(TIF)

S1 Table All plasmids and bacterial strains used in this study. (DOCX)
