# Supplementary figures and images for: Phage resistance at the cost of virulence: Listeria monocytogenes serovar 4b requires galactosylated teichoic acids for InlB-mediated invasion
Source: PLoS Pathog. 2019 Oct 7;15(10):e1008032. doi: 10.1371/journal.ppat.1008032 (PMC6779246; doi:10.1371/journal.ppat.1008032)

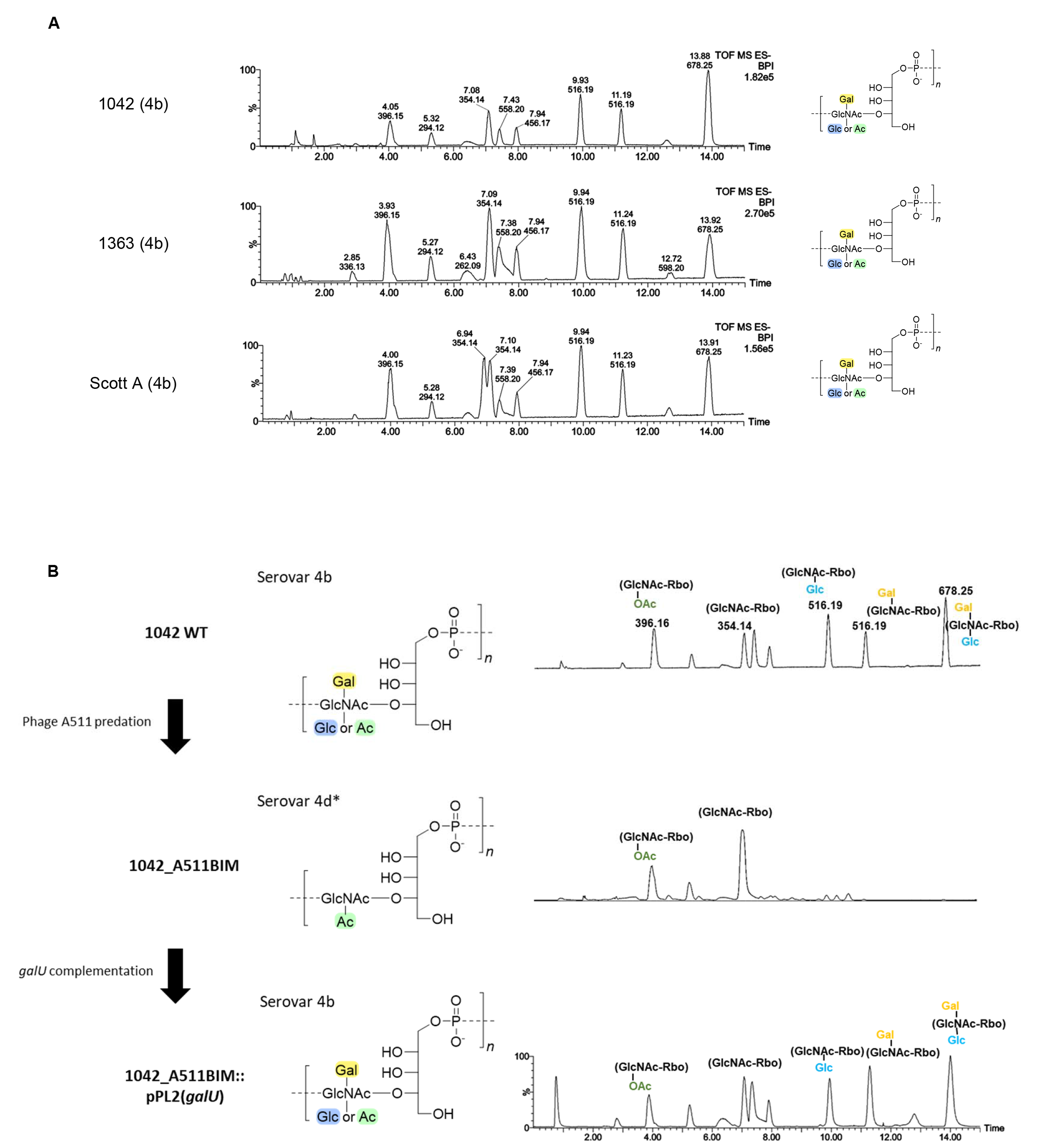

Supplement: S1 Fig — (A) The resulting WTA monomer structures of the indicated strains, as determined by UPLC-MS/MS (data representative of two separate extractions) and the corresponding serovar designation determined via a slide agglutination test. On the chromatograms, peaks are labeled with their corresponding retention time and m/z. (B) The resulting WTA monomer structures of the indicated strains, as determined by UPLC-MS/MS (data representative of two separate extractions) and the corresponding serovar designation determined via a slide agglutination test. On the chromatograms, relevant peaks are labeled with their assigned structures based on the m/z. The dominant peak at 1 min appearing in the galU complemented strain represents the ionized species that elutes without separation, resulting from incomplete de-polymerization. (TIF) [file ppat.1008032.s001.tif]

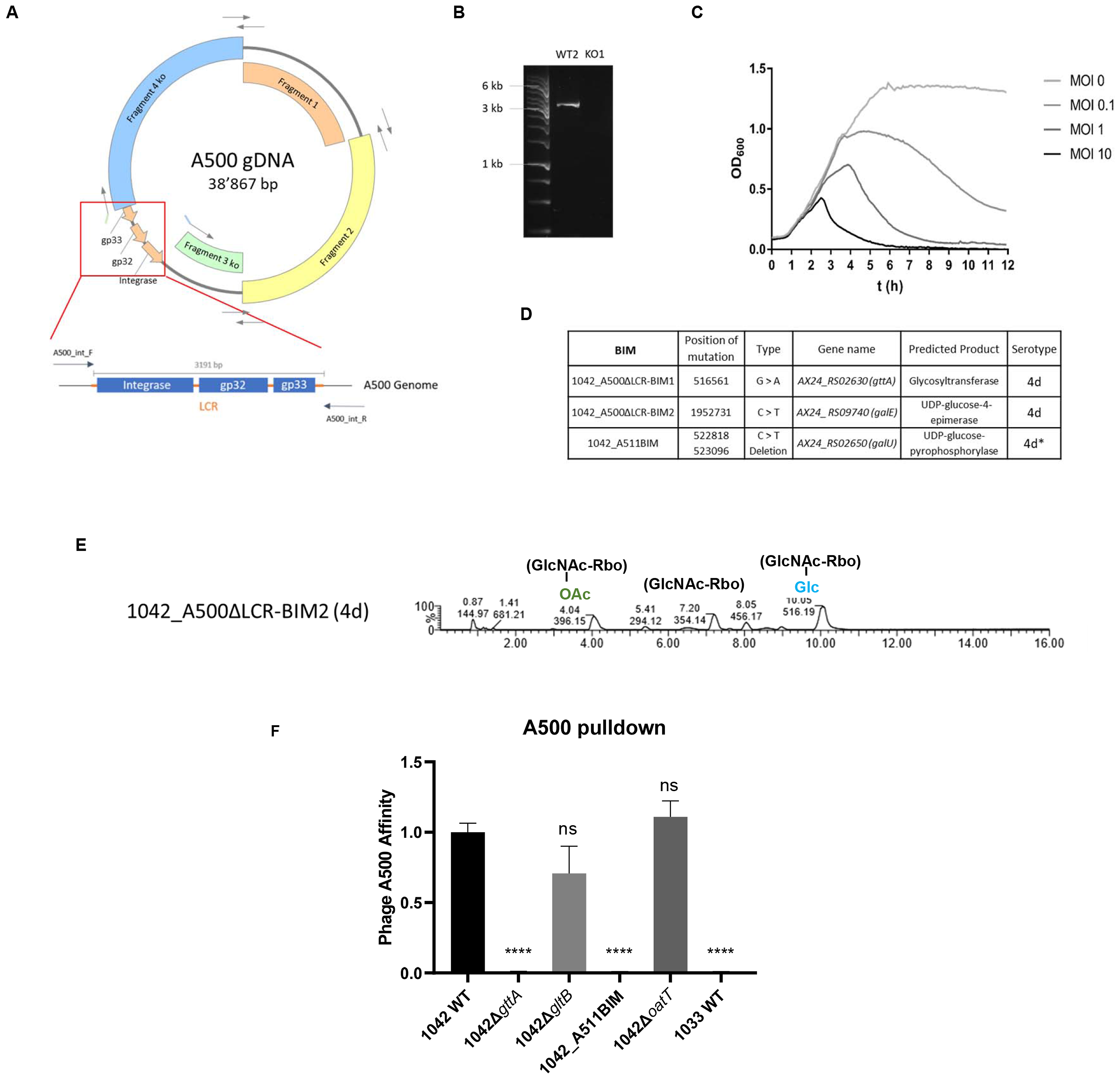

Supplement: S2 Fig — (A) Schematic of the phage A500 genome. Highlighted are the three genes (integrase, gp32, gp33) made up of 3191 bp, which were deleted to produce the mutant phage A500ΔLCR. (B) PCR detection of the A500 genome within the designated phage integration region of 1042 infected with either A500 phage (WT), or A500ΔLCR (KO). The presence of a band corresponds to a positive result for integration i.e. lysogen formation. (C) Growth curves over a 12-hour period of 1042 WT challenged with the indicated MOI of phage A500ΔLCR. (D) Table representing the three BIMs (far left column) discussed in the text, the mutations identified via Illumina re-sequencing, the gene that the mutations fall in, and the resulting phenotype/serotype change. (E) Liquid chromatographic separation and MS-based identification of WTA monomer residues from a select BIM in a 1042 background (harboring a mutation in a gene encoding a UDP-Glucose-epimerase). (F) Phage affinity evaluation using the WT phage A500 against the indicated L. monocytogenes strains, as determined by phage pulldown assays (means normalized to 1042 WT ± SEM, n = 3 for all samples; ****P < 0.0001; ns, not significant relative to 1042 WT, as determined via a one-way ANOVA using 1042 WT as a reference). (TIF) [file ppat.1008032.s002.tif]

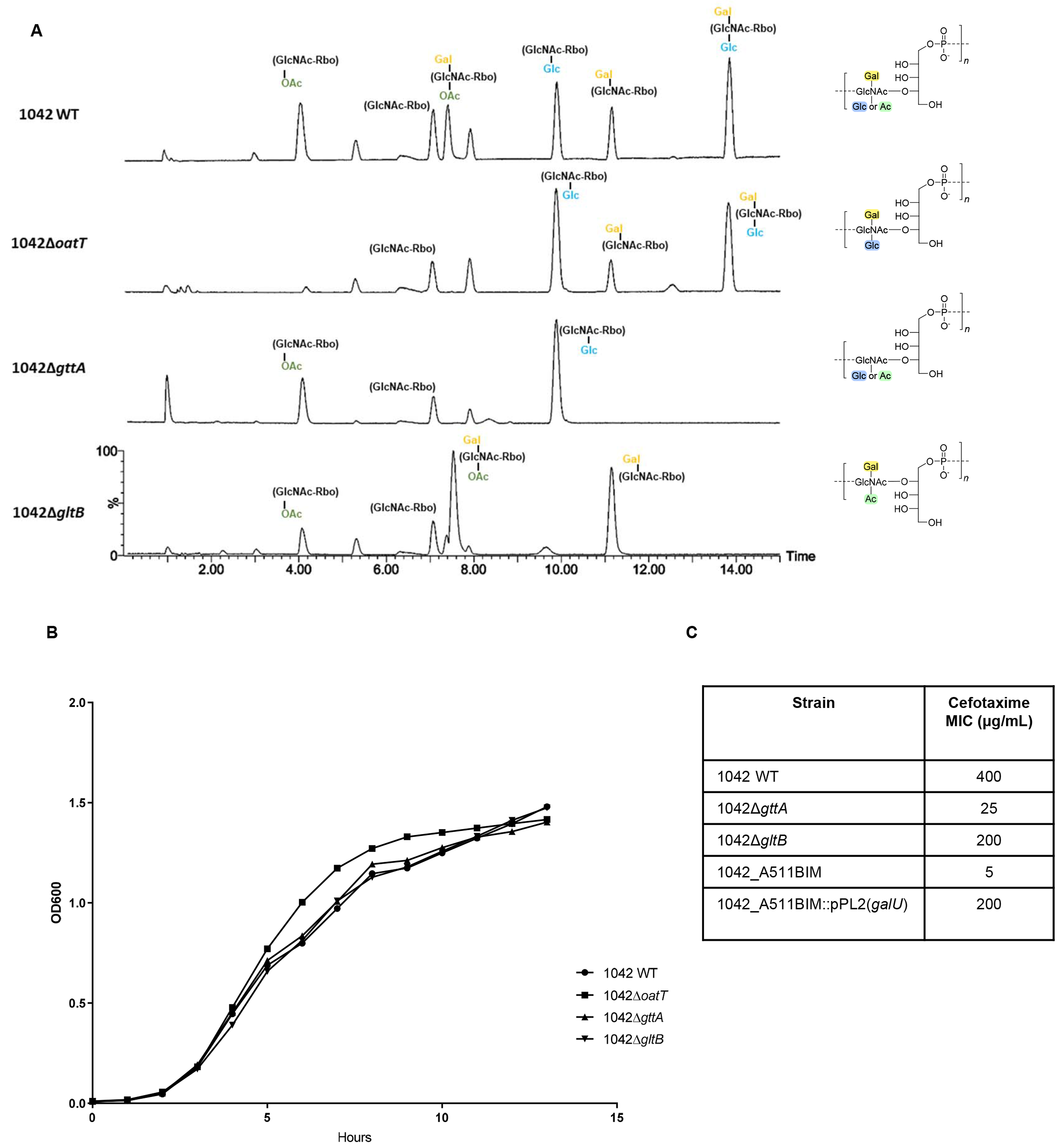

Supplement: S3 Fig — (A) Liquid chromatographic separation and MS-based identification of WTA monomer residues from 1042 and the indicated mutants. The peaks for 1042 WT are labeled with their assigned structures based on the m/z. The chromatograms are aligned on the same time axis to allow for proper comparison. (data is representative of two separate extractions). The dominant peak at 1 min appearing in the 1042ΔgttA mutant strain represents the ionized species that elutes without separation, resulting from incomplete depolymerization. (B) Growth curves (measuring OD600) of the indicated mutants measured over the course of 14 hours (each data point represents n = 3 measurements, error bars were eliminated for visual clarity). (C) Estimated cefotaxime MIC for the indicated mutants (for all, n = 3). (TIF) [file ppat.1008032.s003.tif]

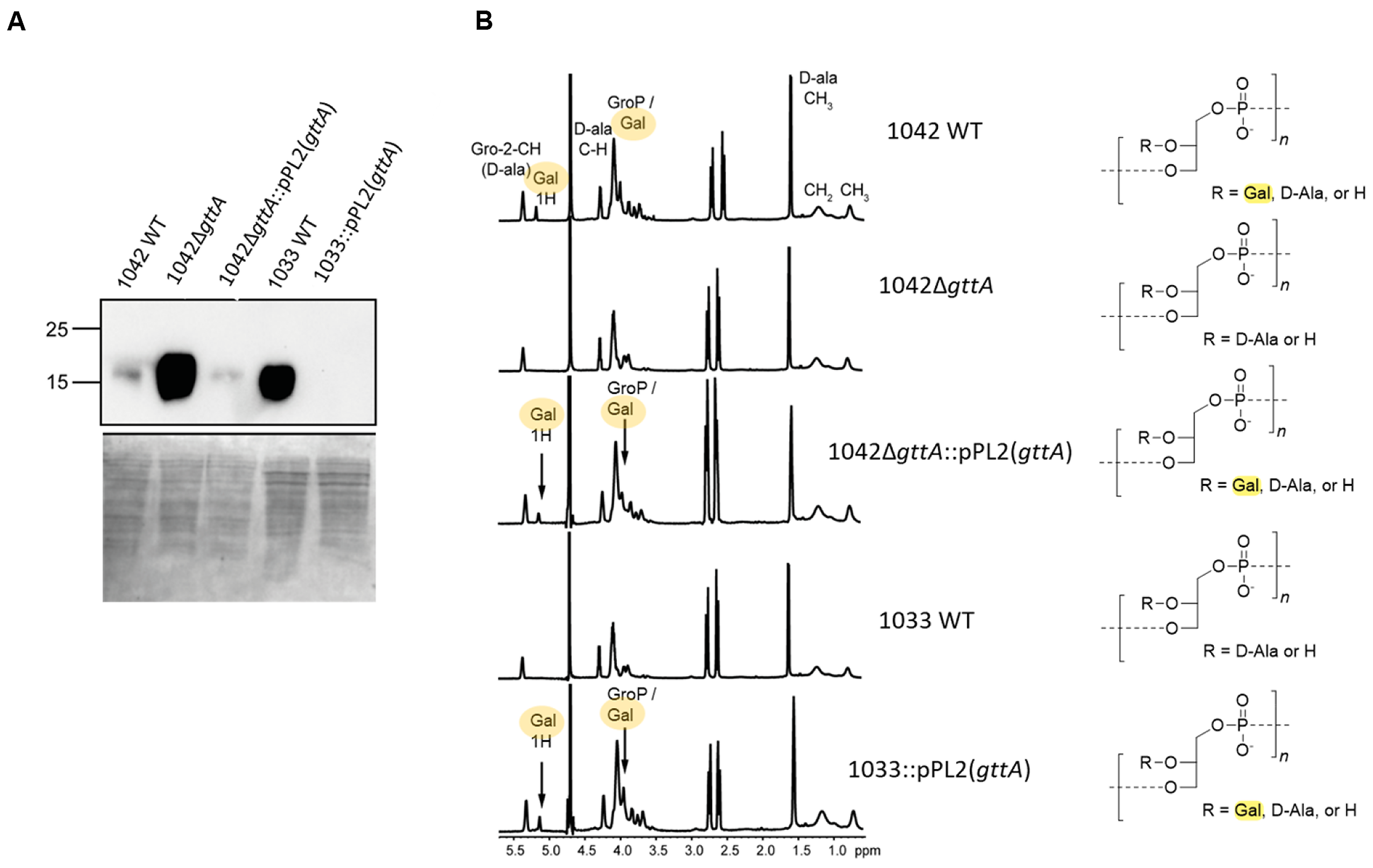

Supplement: S4 Fig — (A) Upper panel: Relative LTA decoration detection as determined by western blot of whole cell extracts using an antibody recognizing undecorated glycerol phosphate (representative of n = 3 blots). Positive signal represents undecorated LTA. Lower panel: Coomassie stain of the same blot to demonstrate equal sample loading. (B) NMR spectra of the repeating units of LTA from the indicated strains. Labeled peaks represent the major protons in the sample, while galactosylated protons are highlighted in yellow. The assigned structures for each strain are indicated on the right. The unlabeled major peaks are derived from residual citrate buffer used during the extraction process. (TIF) [file ppat.1008032.s004.tif]

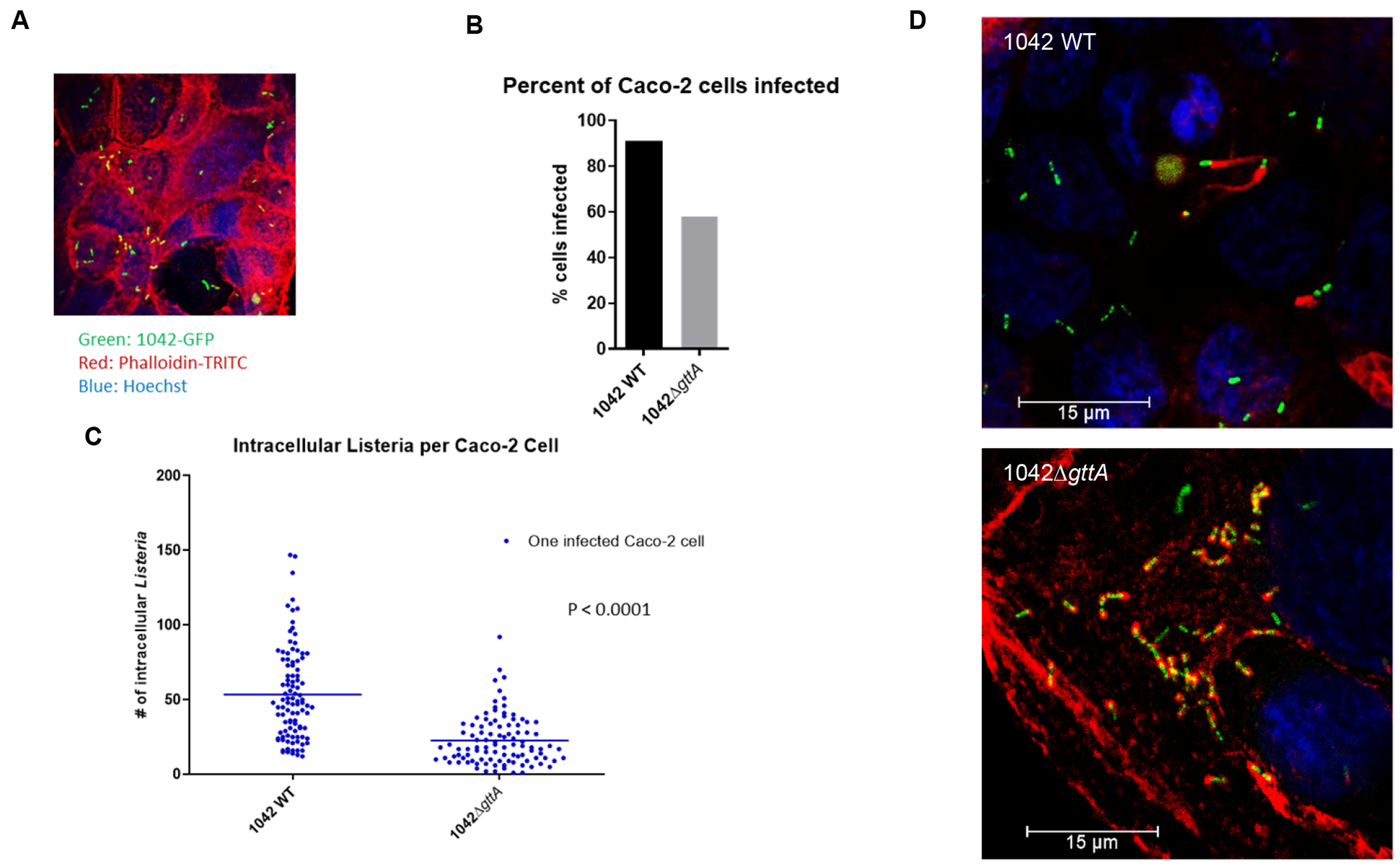

Supplement: S5 Fig — (A) Fluorescence microscopy of 1042-GFP infecting a Caco-2 cell monolayer stained with Phalloidin-TRITC and Hoechst (image is representative of three individual experiments; contrast adjusted for clarity). (B) Percent of Caco-2 cells containing intracellular 1042-GFP or 1042ΔgttA-GFP, as determined via direct observation using fluorescence microscopy as in (A) (numbers are summed from counting fifty cells in two individual experiments). (C) The number of intracellular L. monocytogenes per Caco-2 cell, as determined by fluorescence microscopy as in (A) (each dot represents a single Caco-2 cell, and bars signify the mean intracellular Lmo per cell; numbers were determined by counting fifty cells from two individual experiments; significance was determined by comparing means). Extracellular Listeria were killed and eliminated by gentamicin treatment followed by vigorous washing. (D) Actin tail formation in Caco-2 cells six hours following infection by the indicated strains expressing GFP. Actin stained by phalloidin-TRITC. (TIF) [file ppat.1008032.s005.tif]

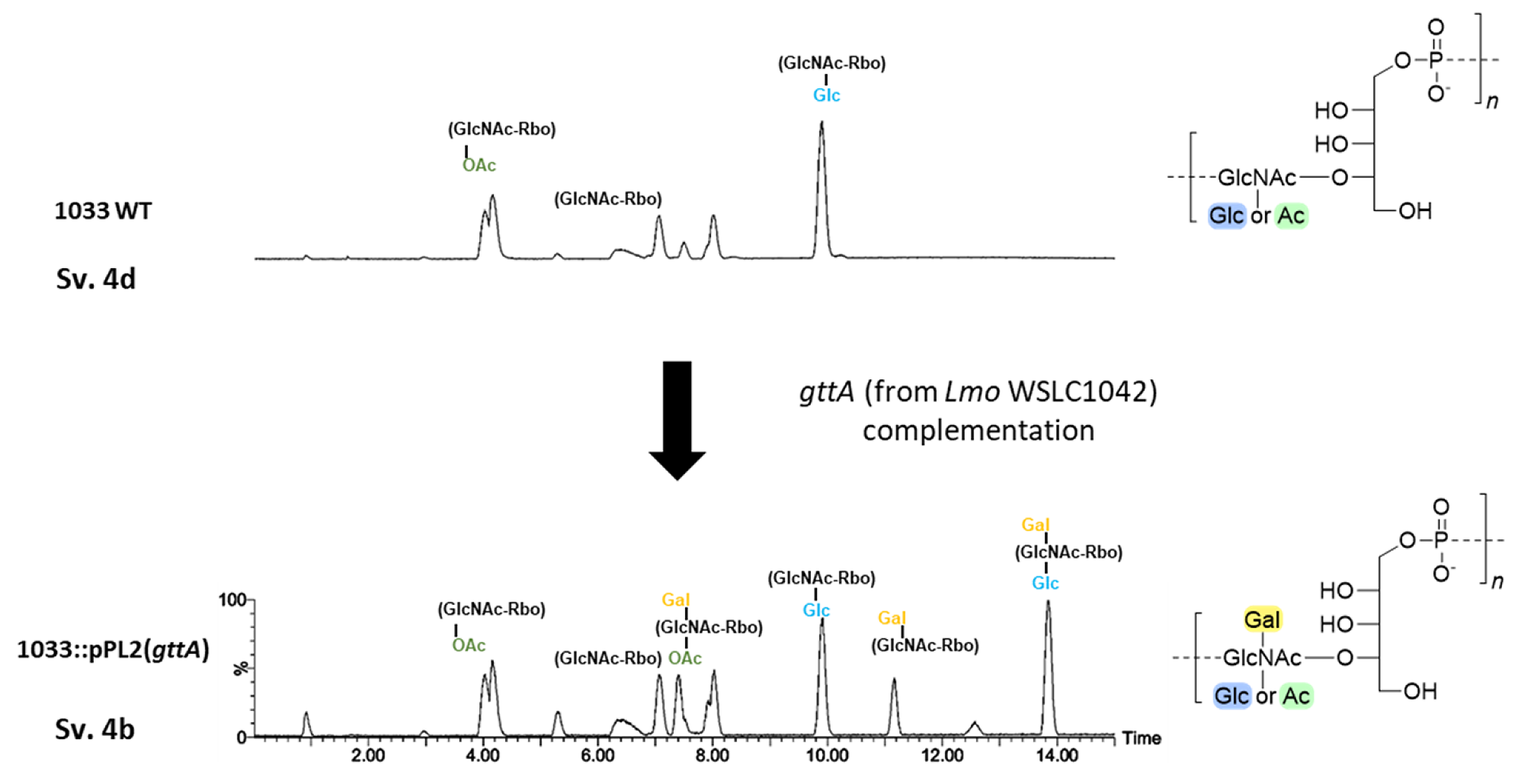

Supplement: S6 Fig — Liquid chromatographic separation and MS-based identification of WTA monomer residues from the indicated strains (left). Relevant peaks are labeled with their assigned structure. The chromatograms are aligned on the same time axis to allow for proper comparison (data is representative of two separate experiments). Predicted WTA monomer structures with the corresponding serovar designation, as determined via a slide agglutination test. (TIF) [file ppat.1008032.s006.tif]

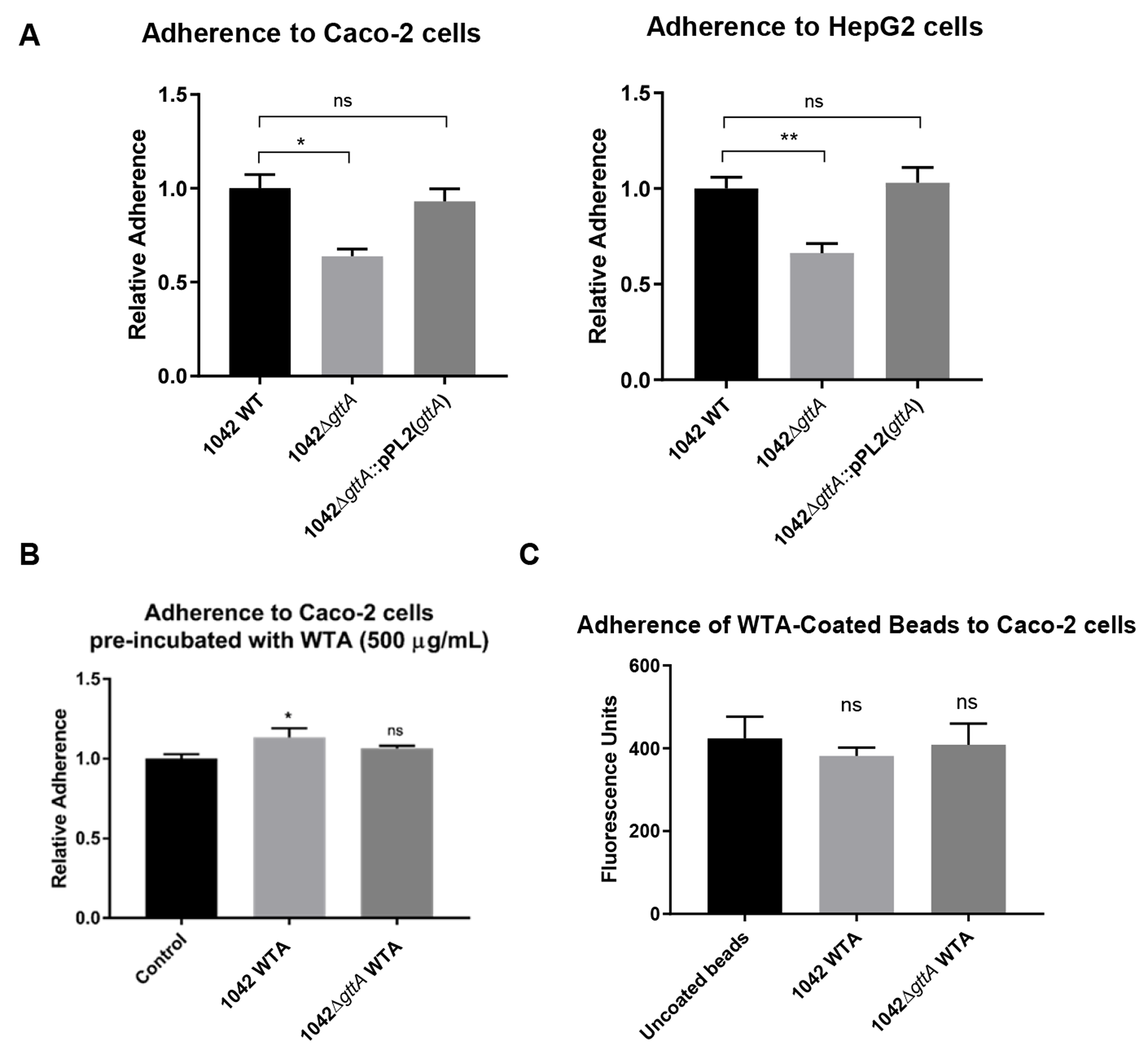

Supplement: S7 Fig — (A) Relative adherence of 1042ΔgttA and 1042ΔgttA::pPL2(gttA) complement compared to 1042 WT, as performed by a 10-minute infection assay in Caco-2 cells (left) and HepG2 cells (right) (means normalized to 1042 WT ± SEM; for both, n = 3; *P<0.05; **P<0.01; ns, not significant). (B) Caco-2 cell adherence of 1042 L. monocytogenes cells incubated in 500 μg/mL of WTA from 1042 or 1042ΔgttA relative to cells incubated in PBS (control) (mean ± SEM; n = 3; *P<0.05; ns = not significant). (C) Adherence of amine-coupled fluorescent latex beads coated with purified WTA from 1042, 1042ΔgttA or uncoated, determined by measuring total fluorescence on a 96-well plate and expressed as arbitrary fluorescence units (ns = not significant compared to uncoated beads control; mean ± SD; n = 8). (TIF) [file ppat.1008032.s007.tif]

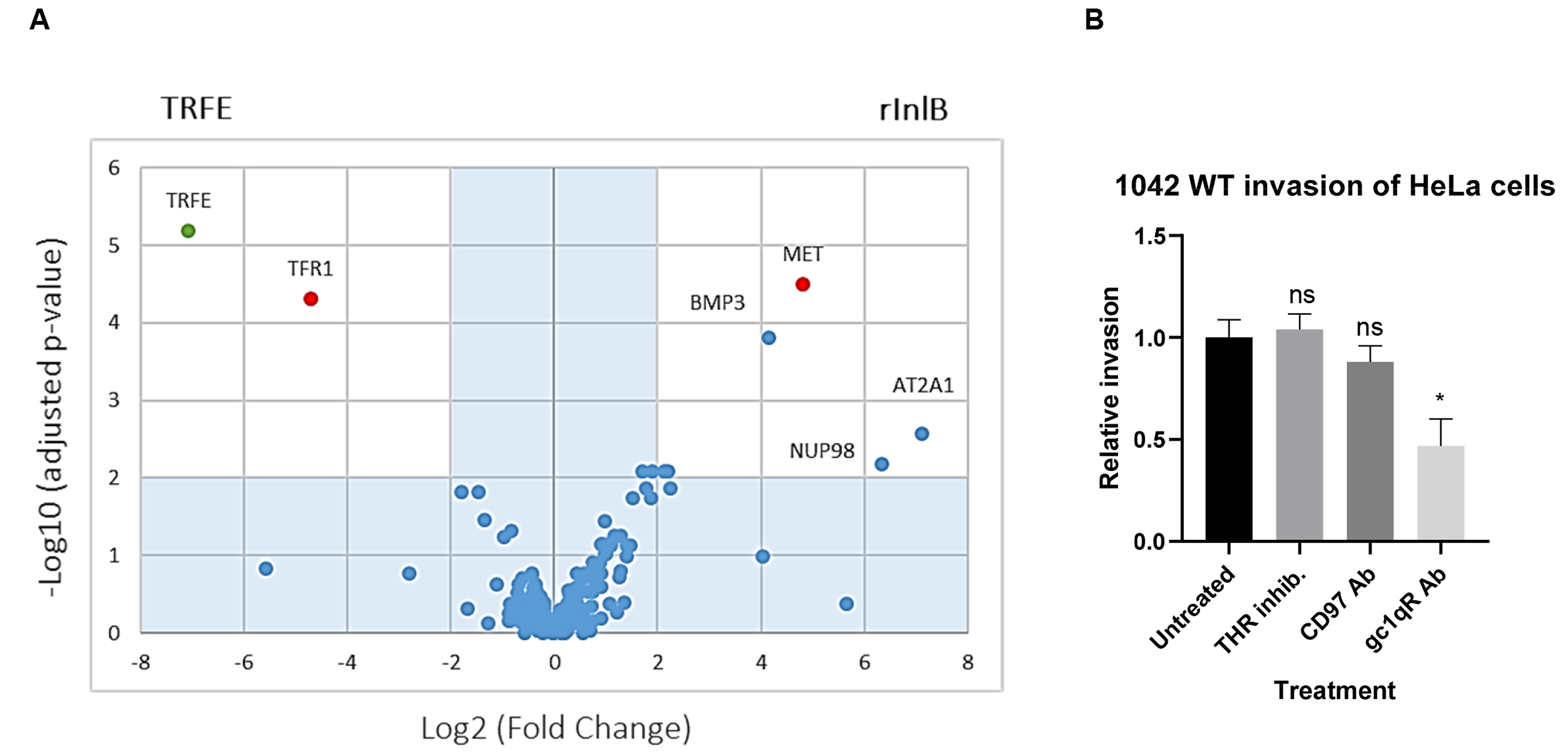

Supplement: S8 Fig — (A) Transferrin (TRFE) was used as a positive control. Results are presented in a volcano plot where relative fold changes of proteins (Log2 scale) are plotted against their respective log-transformed, false-discovery rate (FDR)-adjusted values enabling quick visual identification of proteins that display statistically significant changes. Target receptors are defined as proteins with a fold change greater than 4 and p-value equal to or smaller than 0.01, corresponding to the white space of the plot. Known receptors are shown in red and proteins originating from the ligand are marked in green (data is representative of three individual experiments). (B) Relative invasiveness of 1042 WT determined by infecting HeLa cells for three hours following treatment with a Thyroid hormone receptor antagonist (THR inhibitor.), an anti-CD97 monoclonal antibody, or an anti-gC1qR monoclonal antibody (invasion rate normalized to untreated control ± SEM; n = 3. *P < 0.05; ns, not significant relative to untreated control, as determined by a one-way ANOVA). (TIF) [file ppat.1008032.s008.tif]

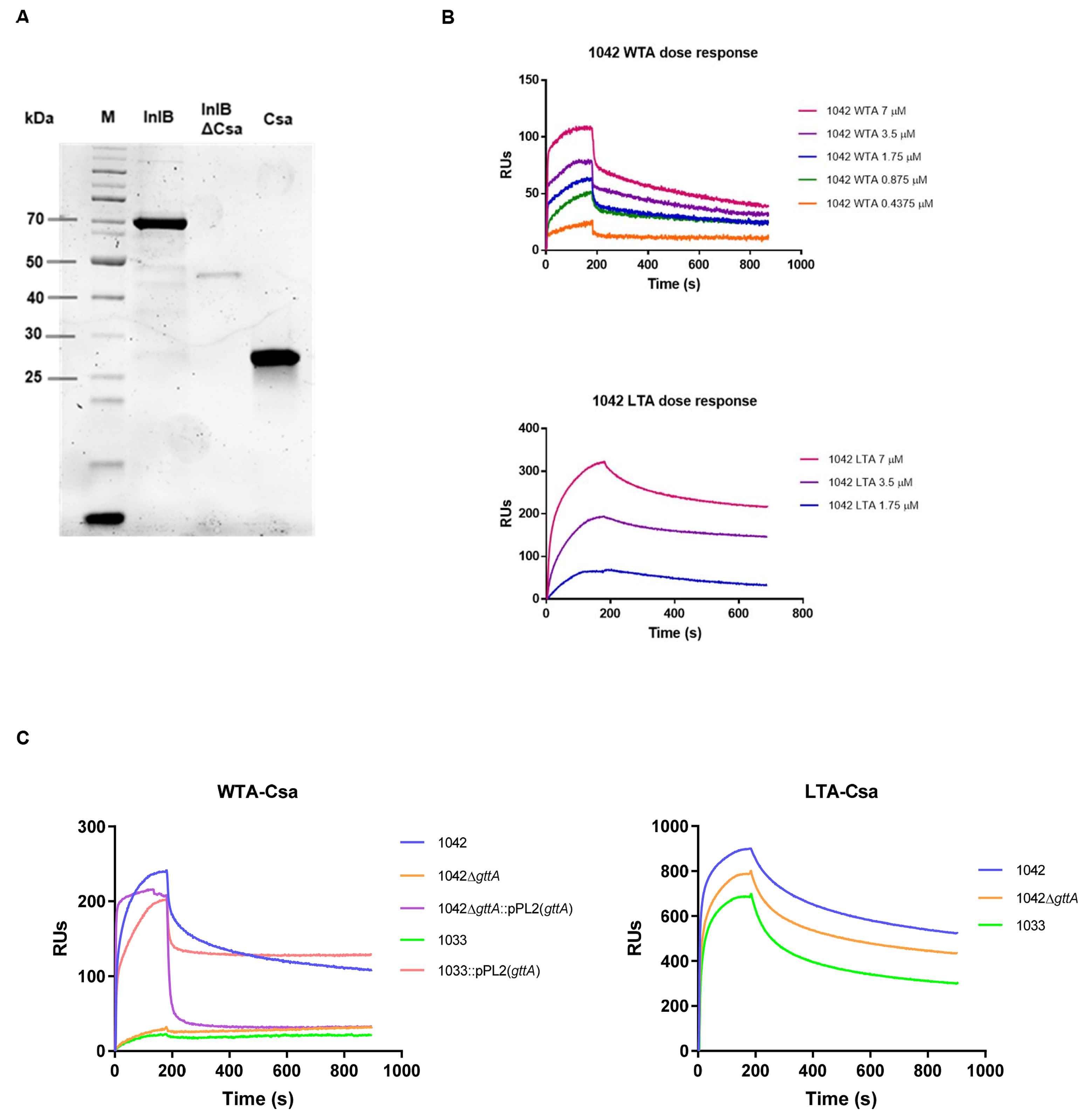

Supplement: S9 Fig — (A) SDS-PAGE analysis of purified N-terminally His-tagged full-length InlB (71.25 kDa), InlBΔCsa (InlB lacking the C-terminal Csa domain containing the GW repeats, 44.78 kDa) and the Csa domain alone (26.48 kDa). (B) TA dose response example titrations of 1042 WTA/LTA (RU: relative units), against immobilized InlB (data used for estimated KA values in Fig 5). (C) Binding kinetics of immobilized Csa (upper) or InlBΔCsa (lower) with WTA (left) or LTA (right) polymers extracted from the indicated strains, as determined by surface plasmon resonance analysis (RU: relative units; data is representative of two individual experiments, each time using newly purified constructs and WTA/LTA extracts). (TIF) [file ppat.1008032.s009.tif]

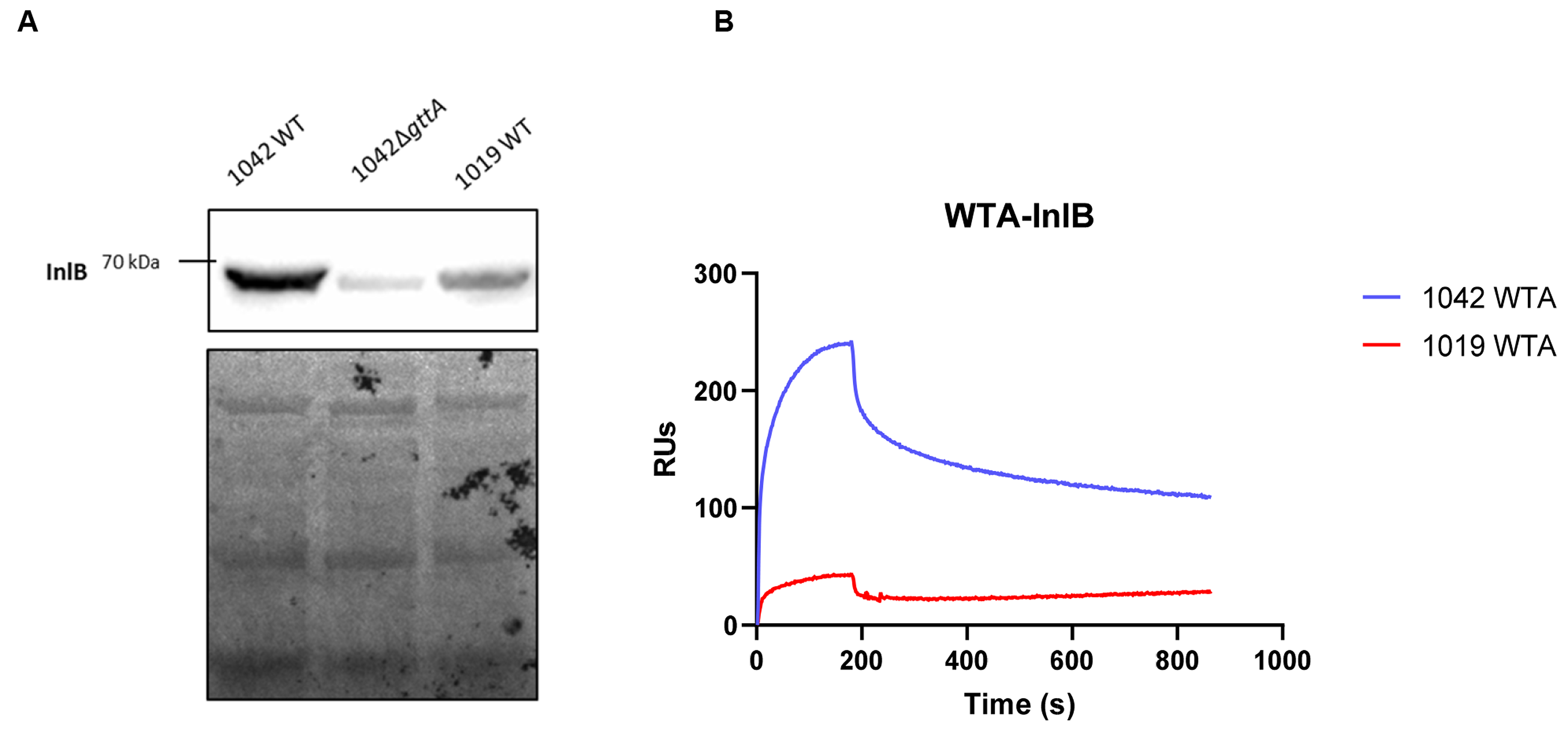

Supplement: S10 Fig — (A) Western blot of total Lmo extracts from the stains 1042, 1042ΔgttA and 1019 (which harbors galactosylated WTA, but at a different orientation from 4b), detected using an anti-InlB antibody. A ponceau stain of the entire nitrocellulose membrane before blotting to demonstrate equal loading is shown in the lower panel. (B) Binding kinetics of immobilized Csa from 1042 with WTA polymers extracted from the indicated strains (compare to S9C), as determined by surface plasmon resonance (RU: relative units). (TIF) [file ppat.1008032.s010.tif]
