# Supplementary material for: Phage resistance at the cost of virulence: Listeria monocytogenes serovar 4b requires galactosylated teichoic acids for InlB-mediated invasion
Source: PLoS Pathog. 2019 Oct 7;15(10):e1008032. doi: 10.1371/journal.ppat.1008032 (PMC6779246; doi:10.1371/journal.ppat.1008032)
Supplement: S1 Table — (DOCX) [file ppat.1008032.s011.docx]

**Table S1.** All plasmids and bacterial strains used in this study

| **Plasmids** | Source |  | Description |
| --- | --- | --- | --- |
| pET302-NT/His | invitrogen |  | N-terminally 6x histidine tagged vector for protein purification |
| pPL2 | Lauer et al., 2002 |  | Integrative shuttle vector for *Listeria* |
| pAUL-A | Chakraborty et al., 1992 |  | Shuttle *Listeria* vector used for knockouts with a temperature-sensitive oriR |
| pHoss1 | Abdelhamed et al., 2015 |  | Shuttle *Listeria* vector used for knockouts with a temperature-sensitive oriR |
| oatT KO | this lab |  | 500 bp flanking regions cloned into the MCS of the shuttle vector pAUL-A for purposes of knockout. In the WSLC 1042 genome, oatT is AX24_RS02625. |
| gttA KO | this lab |  | 500 bp flanking regions cloned into the MCS of the shuttle vector pAUL-A for purposes of knockout. In the WSLC 1042 genome, gttA is AX24_RS02630. |
| gltB KO | this lab |  | 500 bp flanking regions cloned into the MCS of the shuttle vector pHoss1 for purposes of knockout. In the WSLC 1042 genome, *gltB* is AX24_RS11225. |
| inlB KO | this lab |  | 500 bp flanking regions cloned into the MCS of the shuttle vector pHoss1 for purposes of knockout. In the WSLC 1042 genome, *inlB* is AX24_RS14125. |
| ppL2(*gttA*) | this lab |  | *gttA* complementation vector |
| ppL2(*galU*) | this lab |  | *galU* complementation vector |
| ppL2(*gfp*) | this lab |  | GFP knock-in vector |
| ppL3(*rfp*) | this lab |  | RFP knock-in vector |
| pET302-InlB | this lab |  | Full-length InlB (from WSLC 1042) expression vector |
| pET302-InlBΔcsa | this lab |  | Vector expressing InlB lacking csa (GW repeats) |
| pET302-csa | this lab |  | Vector expressing csa only (GW repeats) from InlB |
|  |  |  |  |
| **Strains (*L. monocytogenes*)** | Source | Serotype | Description |
| WSLC 1042 | ATCC 23074 | 4b | ATCC23074™1; GenBank CP007210.1 |
| Scott A | This lab | 4b | WT strain; GenBank CM001159 (Briers et al., 2011) |
| 1363 | This lab | 4b | Outbreak strain: Vacherin mont d'Or cheese, Switzerland |
| WSLC 1018 | ATCC 19118 | 4e | WT strain; GenBank CP013285 |
| WSLC 1019 | ATCC 19116 | 4c | WT strain; GenBank CP013286 |
| WSLC 1020 | ATCC 19114 | 4a | WT strain; GenBank CP013287 |
| WSLC 1033 | ATCC 19117 | 4d | WT strain. GenBank CP013288. Naturally carries a glutamic acid deletion (relative to 1042) at nucleotide position 49 of *gttA*. |
| 1042Δ*oatT* | This lab | 4b | 1042 carrying a deletion in the gene *oatT* |
| 1042Δ*gltB* | This lab | 4b | 1042 carrying a deletion in the gene *gltB* |
| 1042Δ*gttA* | This lab | 4d | 1042 carrying a deletion in the gene *gttA* |
| 1042Δ*gttA*:: pPL2(*gttA*) | This lab | 4b | 1042Δ*gttA* expressing *gttA* via its native promoter from the pPL2 vector (complementation) |
| 1042_A511BIM | This lab | 4(d) | 1042 mutant resistant to phage A511, harboring a SNP at position 522818 (C>T), and a deletion at 523096 leading to a frame shift |
| 1042_A511BIM::pPL2(*galU*) | This lab | 4b | 1042_A511BIM expressing *galU* (AX_RS02650) via its native promoter from the vector pPL2 vector |
| 1042_A500ΔLCRBIM1 | This lab | 4d | 1042 mutant resistant to phage A500ΔLCR harboring a SNP at genome position 516561 (G>A) within gene *gttA* |
| 1042_A500ΔLCRBIM2 | This lab | 4d | 1042 mutant resistant to phage A500ΔLCR harboring a SNP at position 1952731 (C>T) within gene *galE* (AX_RS09740) |
| 1033::pPL2(*gttA*) | This lab | 4b | 1033 expressing *gttA* (from 1042) via its native promoter from the pPL2 vector |
| 1042::pPL2(*gfp*) | This lab | 4b | 1042 expressing *gfp* via its native promoter from the pPL2 integrative vector |
| 1042Δ*gttA*::pPL2(*rfp*) | This lab | 4d | 1042Δ*gttA* expressing *rfp* from the pPL2 integrative vector |
| Rev2 | This lab (P. Studer) |  | EGDe-derived; walled; Pen-inducible conversion to L-form (termed Rev2L); reversible |
| 1042Δ*inlB* | This lab | 4b | 1042 carrying a deletion in the gene *inlB* |
| 1042Δ*inlB*Δ*gttA* | This lab | 4d | 1042 double knockout carrying a deletion in genes *inlB* and *gttA* |
|  |  |  |  |
| **Bacteriophage** | Source |  | Description |
| A500 | Loessner & Busse (1990) |  | *Siphoviridae*; genome size: 38867 bp, temperate |
|  |  |  |  |
| A500ΔLCR | This project |  | Δ*Int*Δ*gp32*Δ*gp3,* genome size: 36296, virulent |
| A511 | Klumpp et al., 2008 |  | *Myoviridae;* genome size: 137619 bp, virulent |
|  |  |  |  |
| **Human cell lines** | Source |  | Description |
| Caco-2 | ATCC HTB-37 |  | Human enterocyte-like colorectal adenocarcinoma cell line |
| HepG-2 | ATCC HB-8065 |  | human hepatocellular carcinoma cell line |
| HeLa | Sigma-Aldrich |  | epitheloid cervix carcinoma |
